# Identification of an Autophagy-Related Signature Based on Whole Bone Marrow Sequencing for the Prognosis and Immune Microenvironment Characterization of Multiple Myeloma

**DOI:** 10.1155/2022/3922739

**Published:** 2022-05-29

**Authors:** Licheng Li, Ting Chen, Jishi Wang, Mengxing Li, Qinshan Li

**Affiliations:** ^1^Clinical Medical College, Guizhou Medical University, 550004 Guiyang, Guizhou Province, China; ^2^Department of Hematology, Affiliated Hospital of Guizhou Medical University, Guizhou Province Institute of Hematology, Guizhou Province Laboratory of Hematopoietic Stem Cell Transplantation Centre, 550004 Guiyang, Guizhou Province, China; ^3^Department of Obstetrics and Gynecology, Prenatal Diagnosis Center, Affiliated Hospital of Guizhou Medical University, 550004 Guiyang, China; ^4^Department of Clinical Biochemistry, School of Clinical Laboratory Science, Guizhou Medical University, 550004 Guiyang, China

## Abstract

Myeloma (MM) is a malignant plasma cell disorder, which is incurable owing to its drug resistance. Autophagy performs an integral function in homeostasis, survival, and drug resistance in multiple myeloma (MM). Therefore, the purpose of the present research was to identify potential autophagy-related genes (ARGs) in patients with MM. We downloaded the transcriptomic data (GSE136400) of patients with MM, as well as the corresponding clinical data from the Gene Expression Omnibus (GEO); the patients were classified at random into two groups in a ratio of 6: 4, with 212 samples in the training dataset and 142 samples in the test dataset. Both multivariate and univariate Cox regression analyses were performed to identify autophagy-related genes. The univariate Cox regression analysis demonstrated that 26 ARGs had a significant correlation with overall survival (OS). We constructed an autophagy-related risk prognostic model based on six ARGs: EIF2AK2 (ENSG00000055332), KIF5B (ENSG00000170759), MYC (ENSG00000136997), NRG2 (ENSG00000158458), PINK1 (ENSG00000158828), and VEGFA (ENSG00000112715) using LASSO-Cox regression analysis to predict risk outcomes, which revealed substantially shortened OS duration in the high-risk cohort in contrast with that in the low-risk cohort. Therefore, the ARG-based model significantly predicted the MM patients' prognoses and was verified in an internal test set. Differentially expressed genes were found to be predominantly enriched in pathways associated with inflammation and immune regulation. Immune infiltration of tumor cells resulted in the formation of a strong immunosuppressive microenvironment in high-risk patients. The potential therapeutic targets of ARGs were subsequently analyzed via protein–drug network analysis. Therefore, a prognostic model for MM was established via a comprehensive analysis of ARGs, through using the clinical models; we have further revealed the molecular landscape features of multiple myeloma.

## 1. Introduction

Multiple myeloma (MM) is a clonal proliferative disease of plasma cells whose characteristics include abnormal immunoglobulin production proliferation of malignant plasma cells into the bone marrow, and bone destruction [1]. Its annual incidence is 4/100,000 people, accounting for approximately 10% of all patients with hematological diseases [2]. Although various treatments including protease inhibitors and immune targeted therapy have been used in clinical practice, however, high recurrence rates and drug resistance rates still affect the prognosis of patients, and the pathogenesis has not been elucidated. Therefore, it is essential to identify new biomarkers for prognosis and understand the pathogenesis of MM.

Autophagy is type II programmed cell death, which is a process of self-digestion and catabolism in cells [3]. Recent evidence indicates that autophagy is a key mechanism for leukemia development and chemotherapy resistance [4]. In addition, autophagy is an essential pro-survival mechanism for medication resistance in MM. The combination therapy of autophagy blockers and traditional anti-MM therapy has enhanced their effects on drug-resistant MM plasma cells, thus making autophagy a novel treatment target [5]. Studies on the function of autophagy in MM are mainly focused on the regulatory mechanisms of several autophagy-related pathways [6]. As far as we know, no research has probed into the potential association between the expression of autophagy-related genes (ARGs) and the MM patients' prognoses.

In the present research, we examined the relationship between ARGs and the prognosis of patients with MM and established a prognostic model for MM. The transcriptome sequencing data of MM patients were obtained from the GEO database, and the patients were classified into the training and test sets. LASSO regression was performed to screen for the key risk factors of patients with MM, construct an ARG-based prognostic risk model, and validate the accuracy of the model in an external verification dataset. The risk model was found to be an independent and effective predictor of prognosis and helped to characterize the MM-specific microenvironment landscape for tumor-infiltrating immune cells and identify potential drug-targeted ARGs. Therefore, the risk model helped to understand the prognostic characteristics and molecular mechanisms of ARGs in MM.

## 2. Materials and Methods

### 2.1. Data Acquisition and Processing

The transcriptome data of 1424 patients were downloaded from a public database, including 354 whole bone marrow samples and pretreated sequencing samples. The raw dataset GSE136400 [7] was acquired from GEO (https://www.ncbi.nlm.nih.gov/geo/), and arrays were processed using the robust multi-array averaging (RMA) algorithm that was performed with the aid of the affy R package [8]. Batch effects were equalized via ComBat analysis using the sva package [9]. The sequencing datasets of patients with MM were obtained from the UCSC Xena database (https://xenabrowser.net/datapages/).

### 2.2. Screening for ARGs

In the Human Autophagy Database (HADb, http://autophagy.lu/clustering/index. html), 209 ARGs were annotated, and 10 genes with expression values of 0 were excluded. The expression profile generated from GSE136400 contained a sum of 199 ARGs.

### 2.3. Screening for ARGs Related to Overall Survival

A total of 354 pretreated sequencing samples of whole bone marrow were randomly divided into two groups in a ratio of 6 : 4, with the training set comprising of 212 patients and the test set comprising of 142 patients. Patients in the training set were screened to elucidate the underlying risk characteristics of the identified ARGs in MM. Eventually, overall survival– (OS–) related ARGs with *P* value < 0.05 were selected utilizing a univariate Cox hazard regression analysis.

### 2.4. Enrichment Analyses of OS-Related ARGs

The Gene Ontology (GO) analysis and the Kyoto Encyclopaedia of Genes and Genomes (KEGG) enrichment analysis were conducted utilizing the ClusterProfiler package [10], which revealed the particular roles of ARGs associated with the OS in MM. The Benjamini–Hochberg with adjusted *P* value < 0.05 was set for the purpose of determining statistical significance.

### 2.5. Characteristics of Molecular Interactions of OS-Related ARGs

To explore the relationship among OS-related ARGs, we employed the STRING database to establish a protein-protein interaction (PPI) network [11] and visualized it using Cytoscape (version: 3.8.0) [12] to calculate the topology level for each module.

TRRUST, an online tool for exploring the relationship between human and mouse transcriptional regulatory networks [13], was used to screen for transcription factors (TFs) associated with key OS-related ARGs.

### 2.6. Establishment and Verification of an ARG-Related Prognostic Model for MM

To prevent these performances to be over-fitted, we used LASSO-Cox regression [14] analysis in the training set to determine key OS-related ARGs for establishing a prognostic model. Multivariable Cox proportional hazard regression analysis was conducted on these ARGs, and variables were gradually selected according to the Akaike information criterion (AIC) [15]. The following is the equation for calculating the final risk score based on the optimized prognostic features:

(1) $$\begin{array}{c} \text{Risk score}=\overset{n}{\underset{i}{\sum }}Coefi\times Ai. \end{array}$$

In this equation, *Coef* denotes the regression coefficient, *i* denotes the ARGs used to construct the signature, *A* denotes the relative expression value of each ARG in the signature, whereas *n* signifies the sum of genes within the signature. Based on the median risk score, patients were classified into 2 cohorts: low- and high-risk cohorts. For the purpose of comparing the two cohorts, the log-rank test and the Kaplan–Meier analysis were performed. The prediction accuracy of the signature was determined using the time-dependent receiver operating characteristic (ROC) curves [16].

To verify the prediction accuracy of the prognostic model, 354 whole bone marrow pretreatment sequencing samples were randomly divided into two groups in a ratio of 6 : 4. The verification group consisted of a sum of 142 patients. The abovementioned equation was employed to derive each patient's risk score, and Kaplan–Meier curves were drawn to reassess differences in prognosis and survival across the two cohorts.

### 2.7. Enrichment Analyses and the Determination of Differentially Expressed Genes (DEGs)

The limma software package [17] was used to identify DEGs in the high- and low-risk groups. GO and KEGG enrichment analyses were conducted with the aid of the ClusterProfiler software package [10], which provides information regarding the GO terms cell component (CC), molecular function (MF), biological process (BP), and KEGG pathways, thus understanding the potential functions of DEGs in MM. In addition, ClustVis [18] was used to cluster the DEGs and generate heat maps.

### 2.8. Gene Set Variation Analysis

As mentioned above, the low- and high-risk cohorts were established based on the median risk score. Gene set variation analysis (GSVA) [19] was used to further confirm the main enrichment pathways of the low- and high-risk cohorts. Nominal *P* value < 0.05 and FDR < 0.25 indicated significant enrichment.

### 2.9. Validation of Prognostic Accuracy of OS-Related ARGs in Clinicopathological Nomograms

The rms software package [20] was used to establish a quantitative prediction technique for the prognosis of patients with MM, and the prognostic features were included in the clinicopathological analyses of the training group. Subsequently, the final model was selected according to the AIC, and calibration curves were charted for the purpose of examining the predictive accuracy regarding the nomogram [21].

### 2.10. Immune Microenvironment Landscape of MM and Possible Immunotherapy Targets for Predicting Prognosis

CIBERSORT was employed to analyze the infiltration levels of 22 distinct immune cells in the low- and high-risk cohorts [22], such as NK cells, plasma cells, B cells, and T cells. The criterion of *P* value < 0.05 was defined as having statistical significance and was used for subsequent analyses as well.

Feasible therapeutic methods for people with MM, immunotherapy, as well as molecular targeted therapy and immunotherapy, have gained increasing attention over the years [23]. We used Spearman correlation analysis to analyze the relationship between the prognostic risk score and treatment goals in clinical practice, and the treatment-related targets were as follows: CTLA-4(ENSG00000163599), VTCN1 (ENSG00000134258), TNFSF18 (ENSG00000120337), TNFSF15 (ENSG00000181634), TNFRSF25 (ENSG00000215788), TNFRSF9 (ENSG00000049249), TNFRSF8 (ENSG00000120949), TMIGD2 (ENSG00000167664), CD276 (ENSG00000103855), PDCD1LG2 (PD-L2)(ENSG00000197646), PDCD1 (PDL1)(ENSSSAG00000045082), LAIR1 (ENSG00000167613), LAG3 (ENSG00000089692), and HAVCR2 (TIM3) (ENSG00000135077).

To screen for potential drug targets of these OS-related ARGs, we employed the NetworkAnalyst 3.0 (https://www.networkanalyst.ca/) to analyze the protein-drug interaction in OS-related ARGs. Information regarding the target and drug content was obtained from the DrugBank (version 5.0, https://go.drugbank.com/) [24].

## 3. Results

### 3.1. Screening of OS-Related ARGs in MM

**Figure 1:**
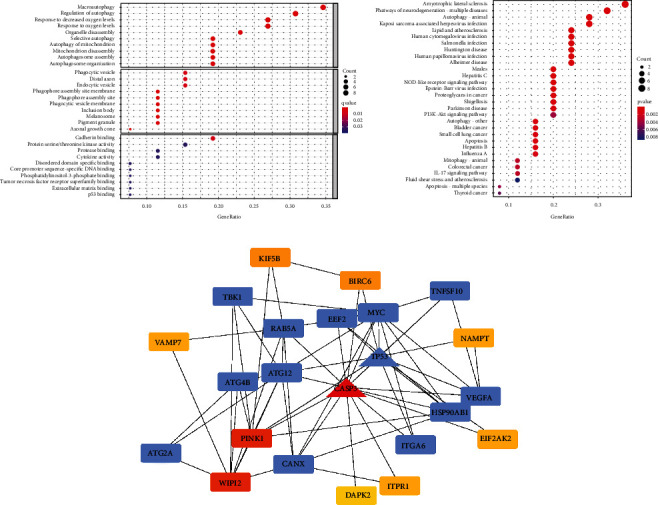
GO terms and KEGG pathways for enrichment analyses of OS-related ARGs in MM (adjusted *P* < 0.05). (a) OS-related ARGs were analyzed for significant enrichment using GO terms. (b) Significant enrichment analysis of OS-related ARGs using the KEGG pathway. (c) Two key modules (CASP3 and TP53) in OS-related ARGs were recognized by analyzing the protein-protein interaction network. The color of nodes in each module indicated their topology scores.

ARGs whose *P* value was less than 0.05 were chosen for the purpose of conducting further investigations by means of a univariate Cox proportional hazard regression analysis so as to determine the relationship between each identified ARG and the MM patients' prognoses. We found that 26 ARGs were correlated with OS (Table 1), and GO analysis of these ARGs revealed that they were mainly enriched in macrophage polarization and regulation of autophagy (Figure 1(a)). In addition, these ARGs were involved in autophagy (animals), apoptosis, PI3K–AKT signaling pathway, and other pathways **(**Figure 1(b)**)**.

To examine the interaction among OS-related ARGs, a PPI network was established, which identified two key modules, namely, CASP3 and TP53, with the highest topology levels (Figure 1(c)). The TP53 module comprised 13 nodes and 26 edges, whereas the CASP3 module consisted of PINK1 and WIPI2 nodes. The regulatory mechanisms of these ARGs may be critical in the pathogenesis of MM.

Furthermore, the TRRUST database (Table S1) was used to identify 42 TFs that regulated these OS-related ARGs and included nuclear TFs (NF-*κ*B1, E2F1, and SP1), histone deacetylase family (HDAC2, HDAC3, and SIRT1), TP53, and NF-*κ*B signal transduction core factors (NF-*κ*B1 and RELA). In addition, important ARG node factors, such as CASP3, MYC, and TP53, were significantly regulated by these TFs.

### 3.2. Screening and Verification of OS-Related ARGs for Prognosis

**Figure 2:**
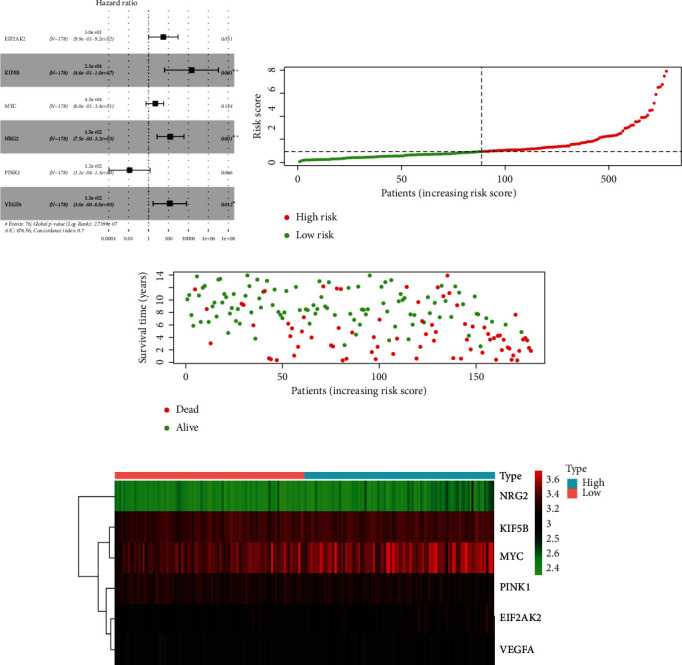
Screening and verification of OS-related ARGs in patients with MM. (a) The risk score was computed using the prognostic model as a basis. (b) Distribution of risk scores in patients. (c) Survival time of high- and low-risk patients with the increasing risk scores. (d) Expression of six OS-related ARGs in patients with MM in the high- and low-risk cohorts.

To avoid potential over-fitting, hub OS-related ARGs were selected for establishing an interaction network using LASSO-Cox regression analysis (Figures S1A and S1B). Six ARGs were identified via the stepwise multivariate Cox proportional hazard regression analysis and were applied to develop a prognostic model for patients with MM (Figure 2(a)). The following is the equation for calculating the risk score of each individual patient:

(2) $$\begin{array}{c} \text{Risk score}=\left[\text{EIF}2\text{AK}2\text{ expression level}*\left(3.4050\right)\right]+\left[\text{KIF}5\text{B expression level}*\left(9.9753\right)\right]+\left[\text{MYC expression level}*\left(1.5109\right)\right]+\left[\text{NRG}2\text{ expression level}*\left(5.0394\right)\right]+\left[\text{PINK}1\text{ expression level}*\left(-4.4047\right)\right]+\left[\text{VEGFA expresion level}*\left(4.8887\right)\right]. \end{array}$$

According to their median risk score, all patients were classified into two cohorts, namely, the high-risk cohort and the low-risk cohort. The death rate of patients increased with the increasing risk score (Figures 2(b) and 2(c)). In addition, six OS-related ARGs, namely, EIF2AK2, KIF5B, MYC, NRG2, PINK1, and VEGFA, were found to have an elevated expression in the high-risk cohort (Figure 2(d) and Figure S1C).

### 3.3. Evaluation of the Prognostic Features of OS-Related ARGs in Patients with MM

**Figure 3:**
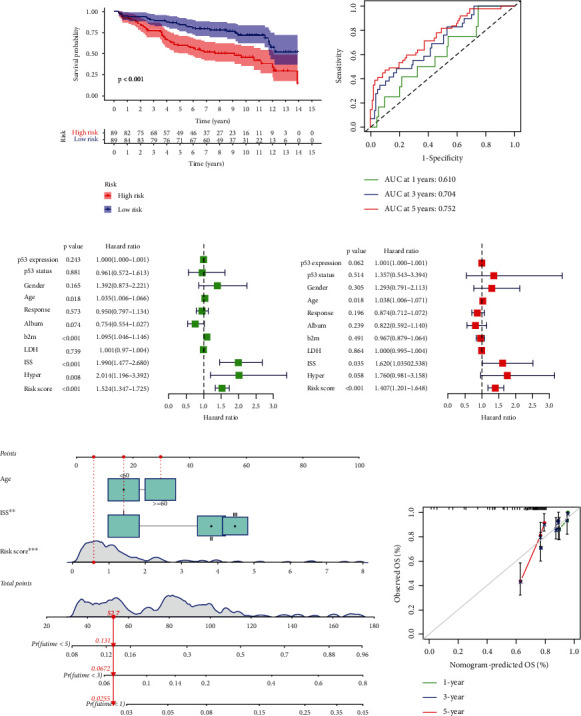
Evaluation of the prognostic features of OS-related ARGs in multiple myeloma patients. (a) Survival status of the low- and high-risk cohorts. (b) ROC curves for anticipating OS over one, three, and five years. (c) Univariate Cox regression analysis of clinical-pathological variables and risk score. (d) Multivariable Cox regression analysis of clinical characteristics and risk score. (e) Nomograms to predict the MM patients' OS over one, three, and five years by combining ISS stage, age, and risk score. (f) The accuracy of the predicted survival rates over one, three, and five years was validated using calibration curves of nomograms. The dashed line indicates an ideal nomogram, whereas the green, blue, and red solid lines refer to the actual utility of the nomogram.

An analysis of Kaplan–Meier survival data was performed for the purpose of verifying the prediction accuracy of the risk signature. The results indicated that the patients' overall survival (OS) in the high-risk cohort was remarkably shorter in contrast with that in the low-risk cohort (*P* < 0.05, Figure 3(a)). Furthermore, the AUC values for anticipating OS over one, three, and five years were shown to be 0.610, 0.704, and 0.752, indicating that the signature had a high predictive power (Figure 3(b)).

A univariate Cox regression analysis was performed for the purpose of examining if the risk signature independently served as a prognostic marker. It was demonstrated that age, *β*_2_-microglobulin level, ISS stage, chromosomal karyotype abnormality, and risk scores were substantially associated with OS (Figure 3(c)). In addition, multivariable Cox regression analysis revealed that after adjusting these clinicopathological factors, the risk signature remained to be a prognostic indicator of MM in an independent manner (Figure 3(d)). Moreover, age and ISS stage were also identified as independently serving as prognostic predictors. Furthermore, the predictive ability of the risk signature was compared among patients with MM according to the risk scores and clinical characteristics. The AUC values for predicting the OS over three, five, and ten years were higher for the risk score than for clinical variables (Figures S2B, S2C, and S2D). However, the AUC value for predicting 1-year OS was lower for the risk score than for some clinical variables (Figure S2A). This finding indicated that the established risk signature is very important for long-term survival prognosis.

To evaluate the signature in a highly accurate manner, a nomogram that combined the risk score, age, and ISS stage was plotted (Figure 3(e)), which demonstrated excellent concordance in predicting OS over one, three, and five years as revealed by calibration plots (Figure 3(f)). The results suggested that the risk score, combined with clinical variables, could anticipate the MM patients' OS in a highly accurate manner.

### 3.4. Enrichment Analysis of ARGs

After observing differences in OS between the low- and high-risk cohorts, we performed GSVA to detect biological functions and related signaling pathways. Humoral immune response and cell cycle pathways exhibited a remarkable enrichment in the low-risk cohort (Figures S3C and S3D), whereas the ubiquitin-mediated proteolysis and endoplasmic reticulum-related degradation (ERAD) pathways were shown to exhibit remarkable enrichment in the high-risk cohort (Figures S3A and S3B). Previous studies have shown that ERAD interacts with the ubiquitin-proteasome pathway and autophagy to reduce protein misfolding or its consequences in a coordinated manner [25]. Therefore, the results indicated that autophagy-related events were associated with high-risk patients with MM.

### 3.5. Validation of the Prognostic Characteristics of the Model

**Figure 4:**
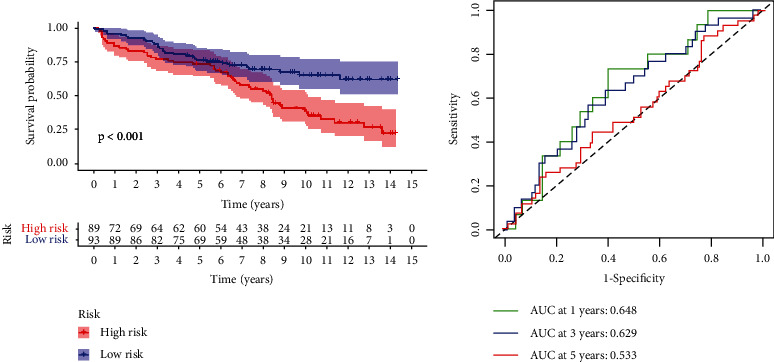
Verification of the prognostic characteristics of OS-related ARGs in the validation set. (a) Kaplan–Meier curve for validation of the prognostic features in the validation set. (b) ROC curve for forecasting OS over one, three, and five years in the validation set.

The risk score formula was used to calculate the risk scores of patients belonging to the low- and high-risk cohorts. The findings revealed that the OS of patients was shorter and their prognosis was worse in the high-risk cohort as opposed to the low-risk cohort (Figure 4(a)). In the validation set, the AUC values for predicting the OS one, two, and three years were 0.648, 0.629, and 0.533, correspondingly (Figure 4(b)). The above data indicated that the risk score independently served as a factor for predicting the MM patients' OS profiles.

### 3.6. Enrichment and Identification of DEGs

**Figure 5:**
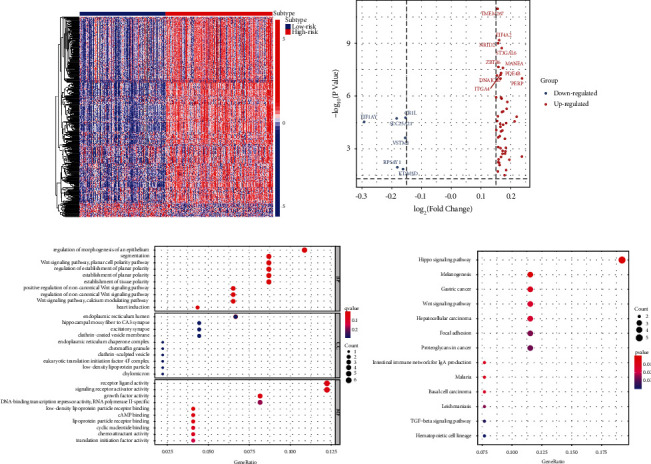
Identification and enrichment of differentially expressed genes (DEGs). (a) DEGs are represented as a heat map. (b) ARGs with significant differential expression. (c) DEGs were subjected to a GO functional enrichment analysis. (d) DEGs were subjected to KEGG pathway analysis.

DEGs were screened in the training and test groups, and their potential functions were assessed. We identified a sum of 56 DEGs in the training set using the limma software package. These DEGs had distinct gene expression patterns in the low- and high-risk cohorts. Of these, 50 genes were upregulated, whereas 6 genes were downregulated (Figures 5(a) and 5(b)). Further, GO analysis illustrated that the ARGs were involved in several biological functions such as regulation of epithelial morphogenesis, endoplasm reticulum lumen, and receptor-ligand activity (Figure 5(c)). KEGG analysis illustrated that the DEGs were primarily involved in the Hippo, Wnt, and TGF-*β* signaling pathways and intentional immune network for IgA production (Figure 5(d)). Considering these crucial biological implications, we inferred that the DEGs played a role in the progression and immunoregulation of MM.

### 3.7. Correlation between Prognostic ARGs and the Immune Microenvironment of MM

**Figure 6:**
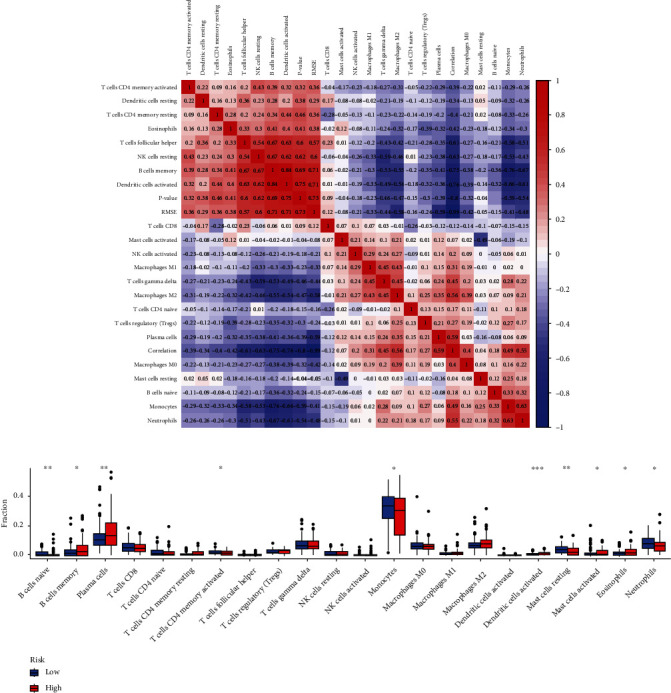
Correlation analysis of the tumor microenvironment and the immune cell infiltration in the low- and high-risk groups. (a) Infiltration scores for each immune cell type were plotted on a heat map by means of row scaling. (b) Histogram of immune cells that have differential infiltration. The red and blue columns indicate the low- and high-risk cohorts.

The interaction between tumor-infiltrating immune cells (TILs) and cancer cells in the tumor microenvironment (TME) is important for cancer progression and drug resistance. Therefore, we assessed the correlation between the OS-related ARG-based risk signature and infiltration of immune cells in TME in the training set and found that the infiltration levels of monocytes, resting mast cells, and neutrophils were remarkably elevated in the low-risk cohort, whereas those of memory B cells, eosinophils, activated mast cells, activated dendritic cells, and plasma cells were increased in the high-risk cohort (Figures 6(a) and 6(b)). These findings illustrated that the risk signature exhibited a significant correlation with TILs present in the microenvironment of MM.

### 3.8. Relationship between the Risk Signature and Immune Checkpoint Therapy

**Figure 7:**
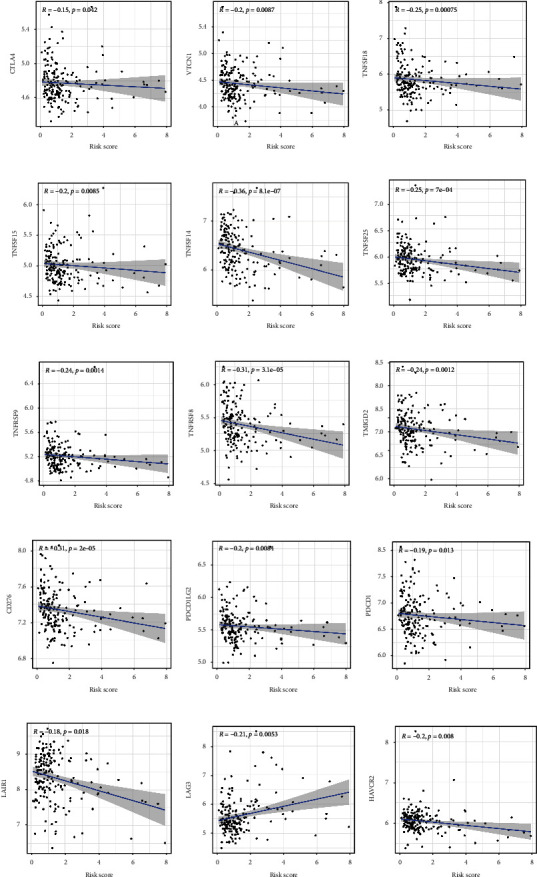
Spearman correlation between risk score and immune checkpoints for tumor-targeted treatment. (a) CTLA-4. (b) VTCN1. (c) TNFSF18. (d) TNFSF15. (e) TNFSF14. (f) TNFRSF25. (g) TNFRSF9. (h) TNFRSF8. (i) TMIGD2. (j) CD276. (k) PDCD1LG2 (PD-L2). (l) PDCD1 (PD1). (m) LAIR1. (n) LAG3. (o) HAVCR2 (TIM3).

Immune checkpoint inhibitors are a new type of targeted immunotherapy, which has recently been used in the preclinical treatment and clinical trials of MM [26]. We used Spearman correlation analysis to examine the relationship between the risk score and various immune checkpoints, including CD274 (PD-L1), CTLA-4, LAG3, and HAVCR2 (TIM3). In addition, differential and correlation analyses were performed for 48 possible checkpoints for tumor-targeted therapy. In addition, the expression of 21 immune factors was found to differ significantly among the risk subgroups (Figures 7 and S4). These results illustrated that the risk score was strongly associated with immune checkpoint genes and tumor immune infiltration. Therefore, patients are more likely to benefit from treatment with immune checkpoints including CTLA-4, PD-L1, LAG3, and HAVCR2.

### 3.9. Identification of Potential Drug Targets of OS-Related ARGs

To screen for potential drug targets of survival-related ARGs, data regarding the protein-drug interactions of these ARGs were obtained from DrugBank and analyzed using NetworkAnalyst. Among these ARGs, the HSP90AB1 gene was identified as a target.

(Table 2 shows drugs with potential targeting of the HSP90AB1 gene in DrugBank).

## 4. Discussion

MM has been identified as the second most prevalent hematological cancer in adults whose characteristics include the buildup of malignant plasma cells within the bone marrow [27]. Emerging therapies have been used in recent years; however, owing to high recurrence and drug resistance rates, their curative effects on patients and the prognosis of patients are poor. In the present research, an accurate prognosis prediction model was established, which may help clinicians in designing individualized treatment strategies for patients. We identified ARGs with a substantial association with the MM patients' OS. Moreover, based on six prognosis-related ARGs, we developed a prognostic risk-score model to predict OS. Analysis of the validation set further suggested that the prognostic model is an independent, stable indicator of OS. Moreover, immune invasion analysis revealed the relationship between the risk score and TILs in the low- and high-risk cohorts, revealing a unique autophagic molecular landscape of the immune microenvironment in MM. Finally, the potential therapeutic targets of MM were identified.

Autophagy is a dynamic and continuous metabolic process. Dysregulation of autophagy is considered a mechanism of survival and drug resistance in many types of cancers. Cancer can upregulate autophagy to promote the survival of cancer cells in TME and increase growth and aggression. Autophagy augments the stemness by degrading ubiquitinated p53, thus relieving the tumor suppressor activity of p53 [28]. Several ATG proteins, as well as their corresponding core complexes, such as LC3 ubiquitin-like binding system, ATG9A transport system, ATG12, autophagy-specific class III PI3K complex, and ULK 1/2 kinase core complex, are the key regulatory factors of tumor occurrence and progression. These regulatory factors targeting autophagy-related key proteins may function as effective intervention approaches for cancer management [6]. In addition, regulators targeting autophagy may represent a promising treatment strategy for myeloma [29]. The use of OS-related ARGs and stratification of high- and low-risk patients help to identify potential prognostic markers for the recurrence and drug resistance of MM.

In this study, 26 ARGs were identified in patients with MM via univariate cox regression analysis, and CASP3 and TP53 were identified as the main modules of these ARGs via protein interaction analysis. Certain ARGs have been involved in the occurrence and progression of cancers and medication resistance to hematologic malignancies. For instance, CASP3 can control the development of AML1-ETO-driven leukemia in a ULK1-dependent manner [30]; TP53 is a well-recognized tumor suppressor gene and mutated TP53 serves as a regulatory factor and target of tumor autophagy [31]. In MM, autophagy is regulated via activation of the p53 pathway and inhibition of CK1 [32]. Furthermore, myc is another well-known oncogene and an autophagy regulator, which can promote the progression and drug resistance of MM [33]. Some other OS-related ARGs, such as DAPK2, EEF2, ITPR1, and VEGFA, have not yet been reported to regulate autophagy in MM; however, they have been confirmed to regulate the progression of other tumors through autophagy [34–37]. However, their specific regulatory mechanisms should be investigated further. In this study, we found that the identified ARGs were significantly enriched in pathways related to the regulation of autophagy, apoptosis, and PI3K–AKT. In addition, PI3K–AKT–mTORC is considered a key factor for the negative regulation of autophagy initiation [38], indicating that these ARGs rely on the PI3K–AKT pathway to regulate autophagy and are involved in the progression of MM.

The establishment of tumor-related prognostic models based on the characteristics of transcriptome expression profiles shows a strong application prospect for risk assessment [39]. In the present research, key OS-related ARGs were identified via LASSO-Cox regression analysis for establishing a risk model. Six ARGs with the best prognostic characteristics were used to construct the model. In both multivariate and univariate Cox regression analyses, adopted for adjustment analysis of clinical variables, the risk score was found to independently serve as a prognostic indicator for MM patients, and the AUC values for predicting OS over one, three, and five years were 0.610, 0.704 and 0.752, correspondingly. Moreover, the AUC value for anticipating 1-year OS was higher for a clinical variable than for the risk score, which suggested that the risk score was extremely significant for long-term survival prognosis. A nomogram integrating ISS stage, age, and risk scores and the calibration curves demonstrated that the model has the potential for accurately anticipating the patients' prognoses. The prediction accuracy of the six OS-related ARGs was similar to those of the five prognostic characteristic genes identified based on RNA sequencing in a study [40]. In addition, the characteristics of the immune microenvironment were explored, and the potential targets of immunotherapy for MM were identified, which may promote the development of drug-based immunotherapy.

Furthermore, DEGs were significantly enriched in the Hippo, Wnt, and TGF-*β* pathways, which are critical signaling pathways that regulate the infiltration of immune cells in TME. For example, the Hippo pathway effector Yap inhibits T cell function and invasion in TME [41], and the Wnt/*β*-catenin pathway is the best-characterized pathway that enhanced the progression of cancer by modulating the tumor immune cycle in the majority of the nodes, such as tumor cells, dendritic cells, and T cells [42]. In addition, blocking the TGF-*β* signal in CD4^+^T cells can reshape TME and inhibit cancer progression [43]. TILs are one of the primary constituents of TME, and the density and types of TILs have marked prognostic associations in multiple cancers [44]. Long-lived plasma cells as well as memory B cells need autophagy in order to survive in MM and perform an integral function in maintaining the survival and medication resistance of tumor cells [45]. In this study, immune infiltration analysis revealed that high-risk patients had high levels of infiltrating eosinophils, activated mast cells, activated dendritic cells, plasma cells, and memory B cells. This finding suggests that a persistently high level of autophagy may be responsible for the intense immunosuppressive microenvironment in MM.

There has been the successful use of immune checkpoint inhibitors in the treatment of hematological cancers and a variety of solid tumors. However, achieving durable response rates in some cancer patients remains challenging because of immune evasion and acquired resistance [46]. In a study, the expression levels of immunosuppressive molecules in eight common tumors exhibited a negative association with the signature-defined risk score, including the classical checkpoints such as PD-L1 and LAG3; however, inhibitors targeting these checkpoints may be less effective in treating patients. Blocking of LAG3 has been shown to significantly enhance T cell activity, kill MM cells, and improve the prognosis of patients with MM [47]. Therefore, continuous activation or inhibition of autophagy may demonstrate the immunosuppressive status of MM patients and their sensitivity to immune-targeted therapy; however, the potential relationship between these two regulatory mechanisms should be investigated further.

Potential strategies to address new drug development challenges and enhance the therapeutic capabilities of existing drugs aim to improve drug target selection and repurpose the existing approved drugs. In this study, the protein-drug interaction analysis of prognosis-related ARGs using the DrugBank database identified only the HSP90AB1 gene as a drug target. There are 14 types of drugs that can target HSP90AB1, and HSP90AB1 has been positively correlated with long non-coding RNA MALAT1 in MM; however, high MALAT1 levels have been correlated with shorter overall progression-free survival [48]. Preclinical studies have shown that HSP90 inhibitors combined with bortezomib enhance anti-MM activity [49]. Therefore, HSP90AB1 is a promising therapeutic target for MM, and further studies should be conducted to develop the next generation of HSP90 inhibitors with higher efficacy and lower toxicity.

The study constructed a characteristic risk prognostic model based on the analysis of autophagy and multiple myeloma patients. We have demonstrated that our models can accurately predict prognosis, but they do have some limitations. We conducted our study entirely using bioinformatics, without an independent external validation cohort. For our study, wet laboratory validation was also necessary to provide additional explanation details. Furthermore, retrospective, published datasets have confirmed that ARGs contribute to disease progression and prospective data are necessary to validate their clinical value and assess their potential clinical relevance.

## 5. Conclusions

In the present research, we established a prognostic model for MM consisting of six ARGs, which was found to independently function as a predictor of OS in both training and verification sets. In addition, we revealed the molecular landscape characteristics of MM, including the cross-correlation among regulatory pathways, TME, and potential drug targets, which were verified based on several aspects. The prognostic model was verified in publicly available retrospective datasets; however, the verification of its potential clinical value requires further investigation using more prospective data, and the specific molecular processes warrant thorough experimental research.

## Figures and Tables

**Table 1:** Overall survival-related ARGs in the MM patients (*P* < 0.05).

Gene	HR	HR.95 L	HR.95H	*P* value
ATG12(ENSG00000145782)	612.39850	9.32780	40205.98970	0.00260
ATG2A(ENSG00000110046)	0.00540	0.00010	0.46110	0.02140
ATG4B(ENSG00000168397)	0.00130	0.00000	0.19580	0.00940
BIRC6(ENSG00000115760)	2.54180	4.41450	26758.17720	0.01980
CANX(ENSG00000127022)	200.47830	8.68320	4628.64190	0.00090
CASP3(ENSG00000164305)	20.79620	1.34550	321.43170	0.02980
CD46(ENSG00000117335)	23.82250	3.03330	187.09140	0.00260
DAPK2(ENSG00000035664)	0.01220	0.00040	0.37550	0.01170
EEF2(ENSG00000167658)	7.90060	1.07900	8.50690	0.04610
EIF2AK2(ENSG00000055332)	27.53830	12.32600	87.67210	0.00050
HSP90B(ENSG00000096384)	44.34080	3.16210	621.76850	0.00490
ITGA6(ENSG00000091409)	6.59830	1.14420	38.05000	0.03480
ITPR1(ENSG00000150995)	34.00590	3.48940	331.40550	0.00240
KIF5B(ENSG00000170759)	878.23420	5.24960	146924.13170	0.00950
KLHL24(ENSG00000114796)	24.95400	1.99070	312.80390	0.01260
MYC(ENSG00000136997)	25.51130	3.30050	197.18840	0.00190
NAMPT(ENSG00000105835)	9.10870	1.32960	62.40010	0.02440
NRG2(ENSG00000158458)	21.50710	1.36870	337.94100	0.02900
PINK1(ENSG00000158828)	0.00390	0.00000	0.33360	0.01450
RAB5A(ENSG00000144566)	200.71470	3.04860	13214.65620	0.01310
TBK1(ENSG00000183735)	77.82610	1.16250	5210.22020	0.04230
TNFSF10(ENSG00000121858)	8.66860	1.29880	57.85890	0.02580
TP53(ENSG00000141510)	23.00740	1.40580	376.54580	0.02790
VAMP7(ENSG00000124333)	38.13020	1.74430	833.50030	0.02070
VEGFA(ENSG00000112715)	268.95920	8.52410	8486.43260	0.00150
WIPI2(ENSG00000157954)	0.00110	0.00000	0.20250	0.01060

**Table 2:** Drugs with potential targeting of HSP90AB1 gene in DrugBank.

Id	Label
DB02424	Geldanamycin
DB02754	9-Butyl-8-(3,4,5-Trimethoxybenzyl)-9 h-Purin-6-amine
DB03758	Radicicol
DB05134	CNF1010
DB06070	SNX-5422
DB07594	4-[4-(2,3-DIHYDRO-1,4-BENZODIOXIN-6-YL)-3-METHYL-1H-PYRAZOL-5-YL]-6-ETHYLBENZENE-1,3-DIOL
DB07877	8-(6-BROMO-BENZO[1,3]DIOXOL-5-YLSULFANYL)-9-(3-ISOPROPYLAMINO-PROPYL)-ADENINE
DB08045	4-{4-[4-(3-AMINOPROPOXY)PHENYL]-1H-PYRAZOL-5-YL}-6-CHLOROBENZENE-1,3-DIOL
DB08153	(5E)-14-CHLORO-15,17-DIHYDROXY-4,7,8,9,10,11-HEXAHYDRO-2-BENZOXACYCLOPENTADECINE-1,12(3H,13H)-DIONE
DB08292	(5Z)-12-CHLORO-13,15-DIHYDROXY-4,7,8,9-TETRAHYDRO-2-BENZOXACYCLOTRIDECINE-1,10(3H,11H)-DIONE
DB08293	(5E)-12-CHLORO-13,15-DIHYDROXY-4,7,8,9-TETRAHYDRO-2-BENZOXACYCLOTRIDECINE-1,10(3H,11H)-DIONE
DB08346	(5Z)-13-CHLORO-14,16-DIHYDROXY-3,4,7,8,9,10-HEXAHYDRO-1H-2-BENZOXACYCLOTETRADECINE-1,11(12H)-DIONE
DB08464	METHYL 3-CHLORO-2-{3-[(2,5-DIHYDROXY-4-METHOXYPHENYL)AMINO]-3-OXOPROPYL}-4,6-DIHYDROXYBENZOATE
DB08465	2-(3-AMINO-2,5,6-TRIMETHOXYPHENYL)ETHYL 5-CHLORO-2,4-DIHYDROXYBENZOATE

## Data Availability

The manuscript contains the actual data provided in the present research; additional inquiries can be sent to the respective authors.

## Supplementary Materials

Supplementary 1 Supplementary Figure 1: LASSO regression analysis is performed to identify crucial variables. (a) 1000-fold cross-validation for variable selection in LASSO regression. (b) Coefficients of LASSO regression for significant autophagy-related genes. Each curve corresponds to a gene involved in autophagy. (c) Six model genes were expressed in low- and high-risk cohorts (*P* < 0.05).

Supplementary 2 Supplementary Figure 2: different multiple receivers operating characteristic (ROC) curves for risk score and clinical factors. (a) Multiple receivers operating characteristic curves for estimating one-year OS. (b) Multiple ROC curves for anticipating the OS over three years. (c) Multiple ROC curves for anticipating the OS over 5 years. (d) Multiple ROC curves for anticipating the OS over 10 years.

Supplementary 3 Supplementary Figure 3: analysis of the gene set enrichment regarding the autophagy-related signature in low- and high-risk cohorts. (a, b) Genes enriched in the high-risk cohort. (c, d) Genes enriched in the low-risk cohort.

Supplementary 4 Supplementary Figure 4: the tumor immune microenvironment analysis in the low- and high-risk cohorts. (a) Differential expression of 21 types of immune checkpoints in the low- and high-risk cohorts. (b) Analysis of the correlation between the risk score and multiple immune checkpoints.

Supplementary 5 Supplementary Table 1: identification of the transcriptional regulators of OS-associated ARGs.

Supplementary 6 Supplementary Table 2: characteristics of all patients.
