# Supplementary figures and images for: Identification of an Autophagy-Related Signature Based on Whole Bone Marrow Sequencing for the Prognosis and Immune Microenvironment Characterization of Multiple Myeloma
Source: J Immunol Res. 2022 May 29;2022:3922739. doi: 10.1155/2022/3922739 (PMC9169202; doi:10.1155/2022/3922739)

A

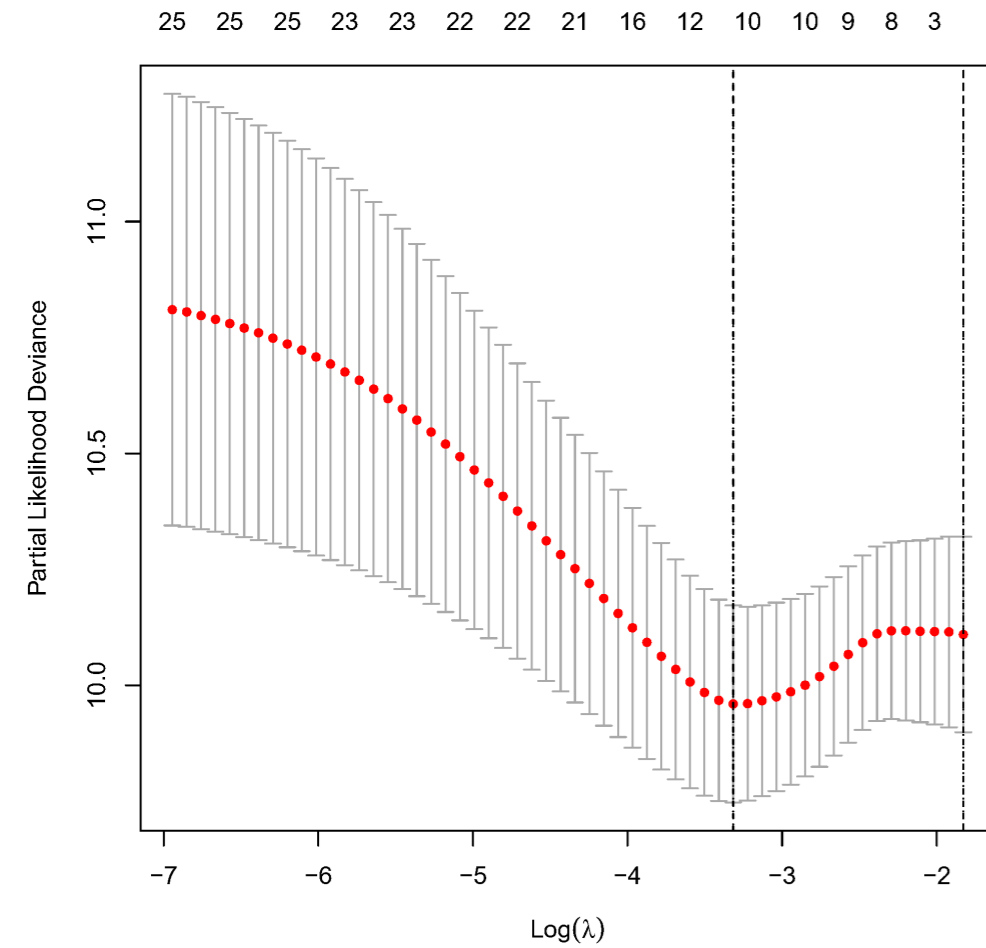

B

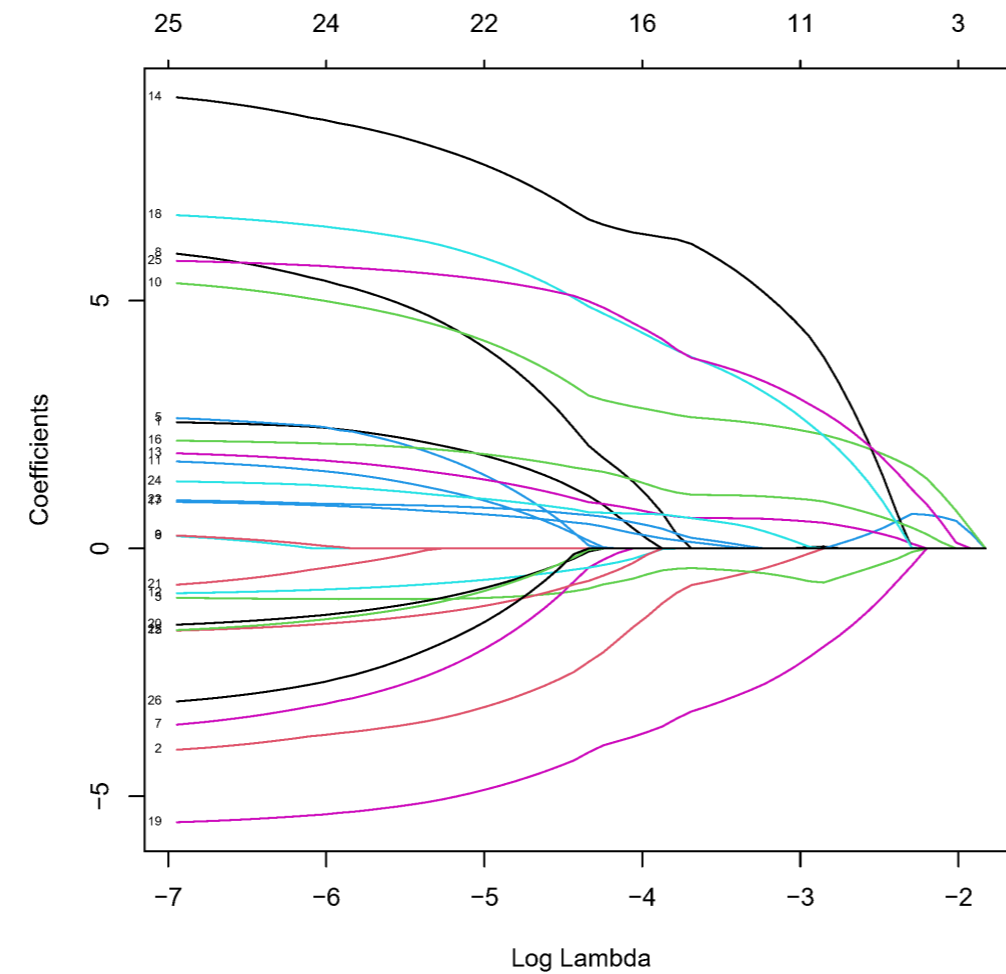

C

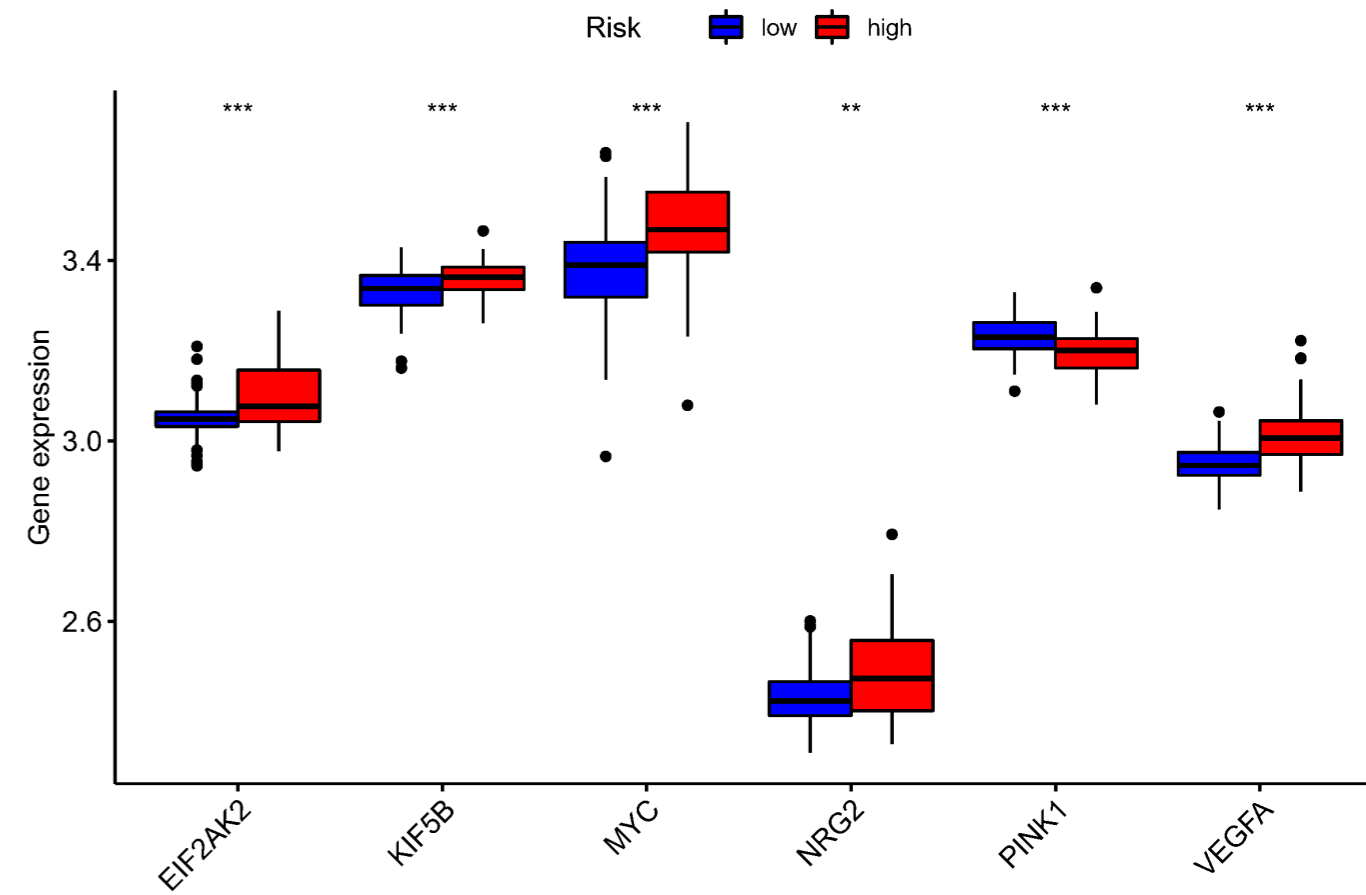

Supplement: Supplementary 1 — Supplementary Figure 1: LASSO regression analysis is performed to identify crucial variables. (a) 1000-fold cross-validation for variable selection in LASSO regression. (b) Coefficients of LASSO regression for significant autophagy-related genes. Each curve corresponds to a gene involved in autophagy. (c) Six model genes were expressed in low- and high-risk cohorts (P < 0.05). [file 3922739.f1.pdf]

A

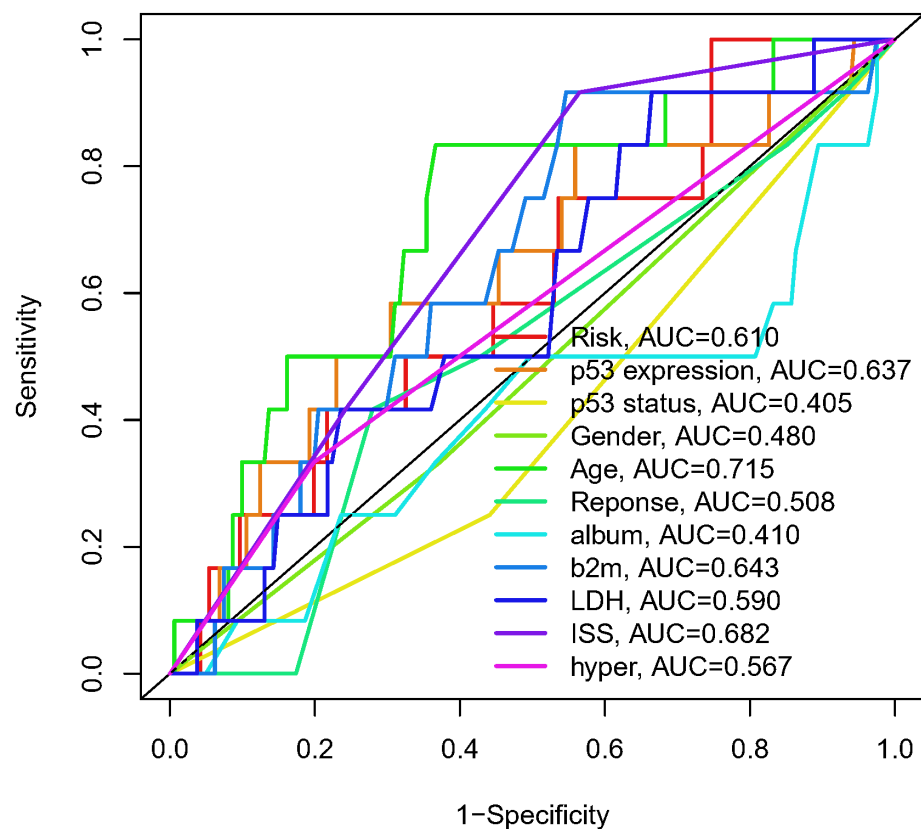

B

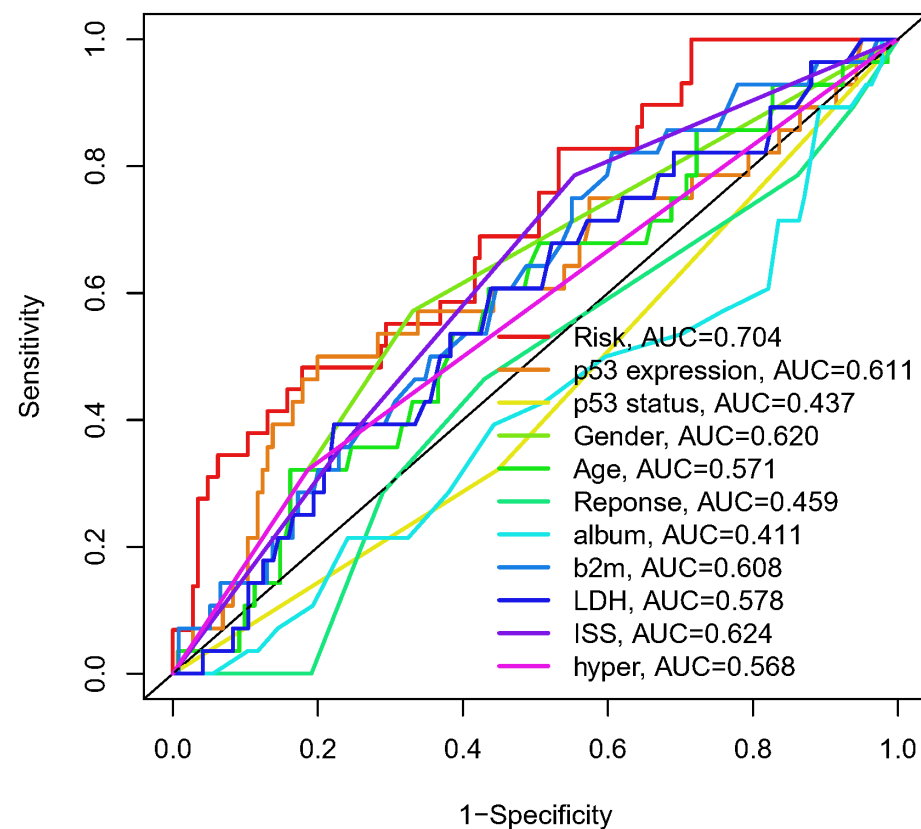

C

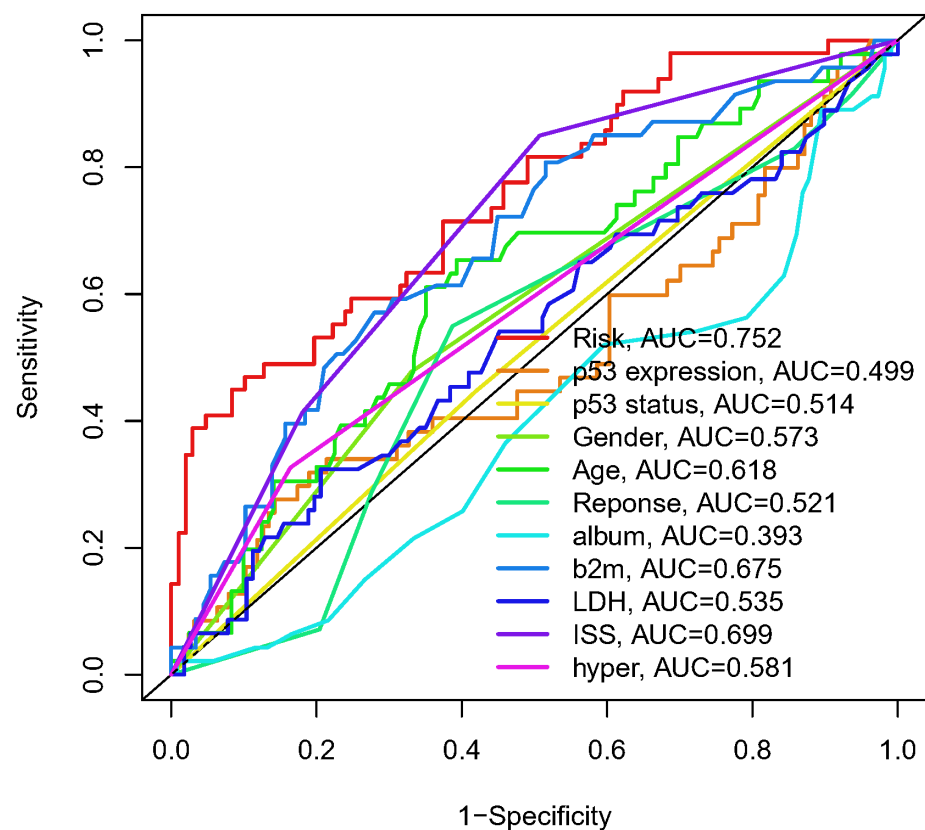

D

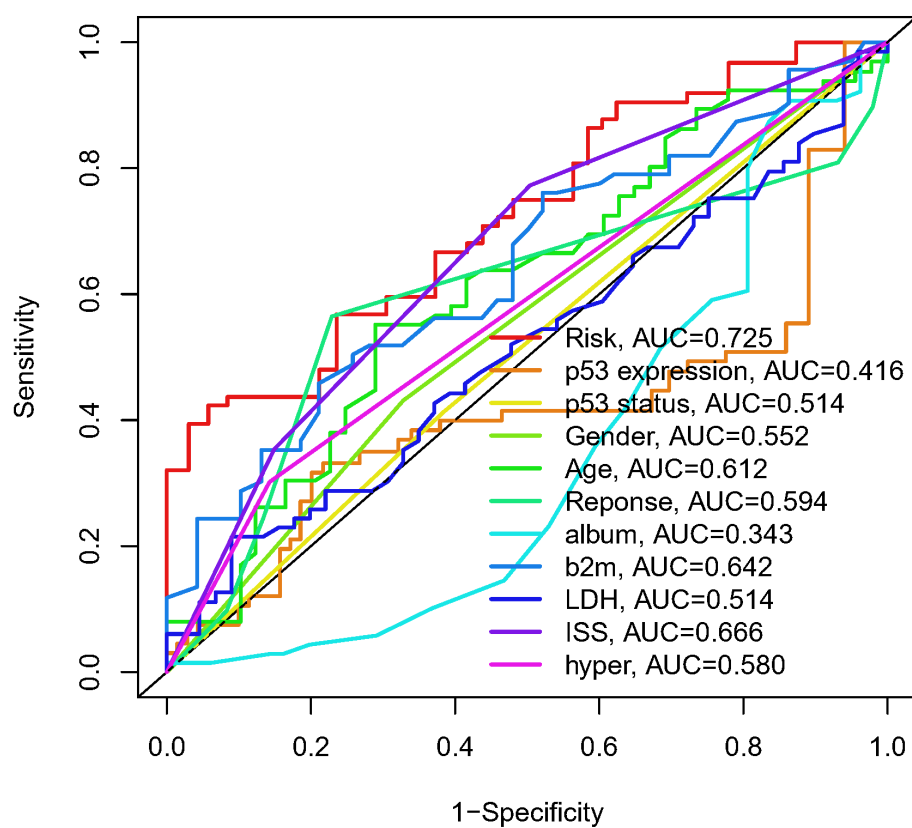

Supplement: Supplementary 2 — Supplementary Figure 2: different multiple receivers operating characteristic (ROC) curves for risk score and clinical factors. (a) Multiple receivers operating characteristic curves for estimating one-year OS. (b) Multiple ROC curves for anticipating the OS over three years. (c) Multiple ROC curves for anticipating the OS over 5 years. (d) Multiple ROC curves for anticipating the OS over 10 years. [file 3922739.f2.pdf]

A

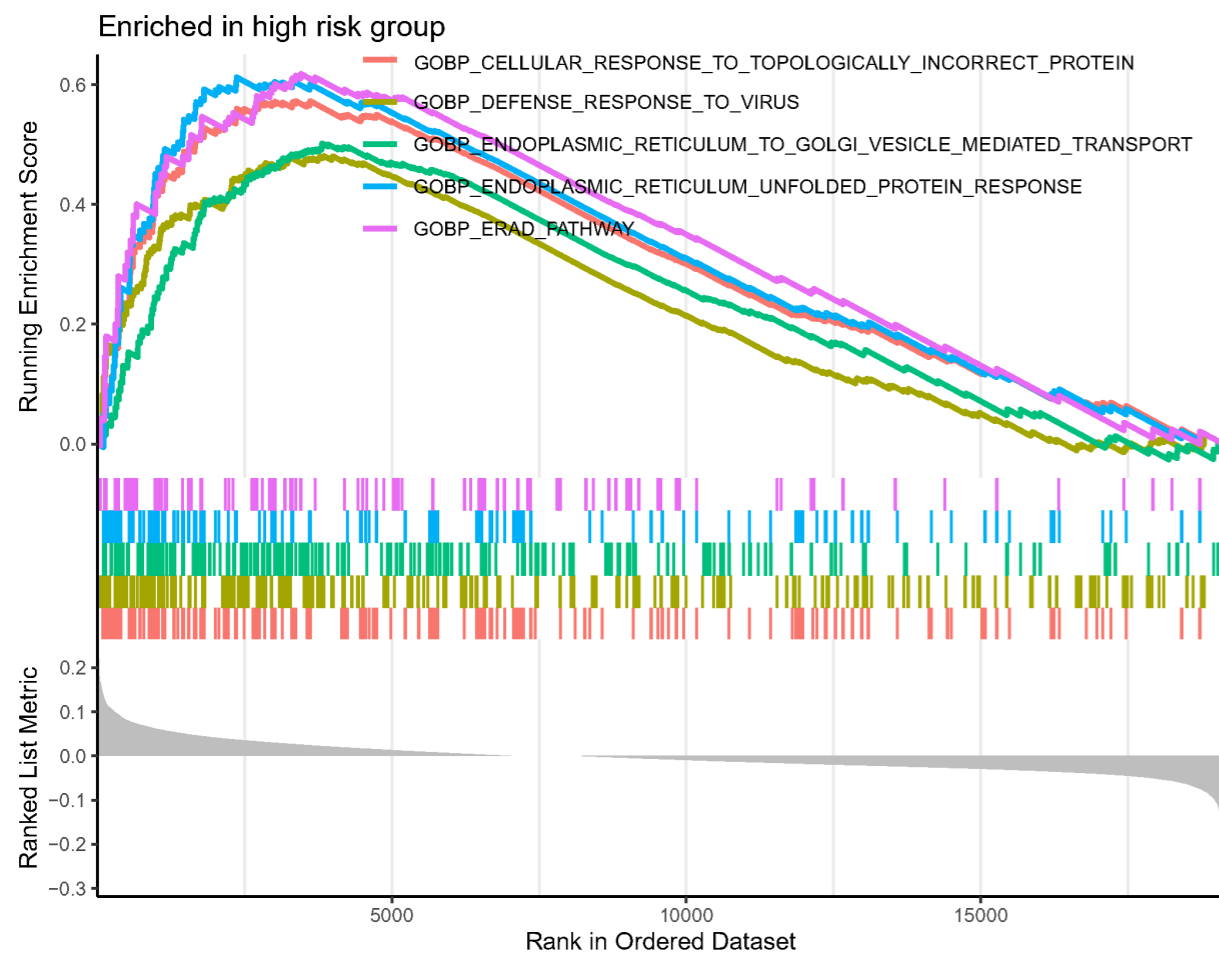

B

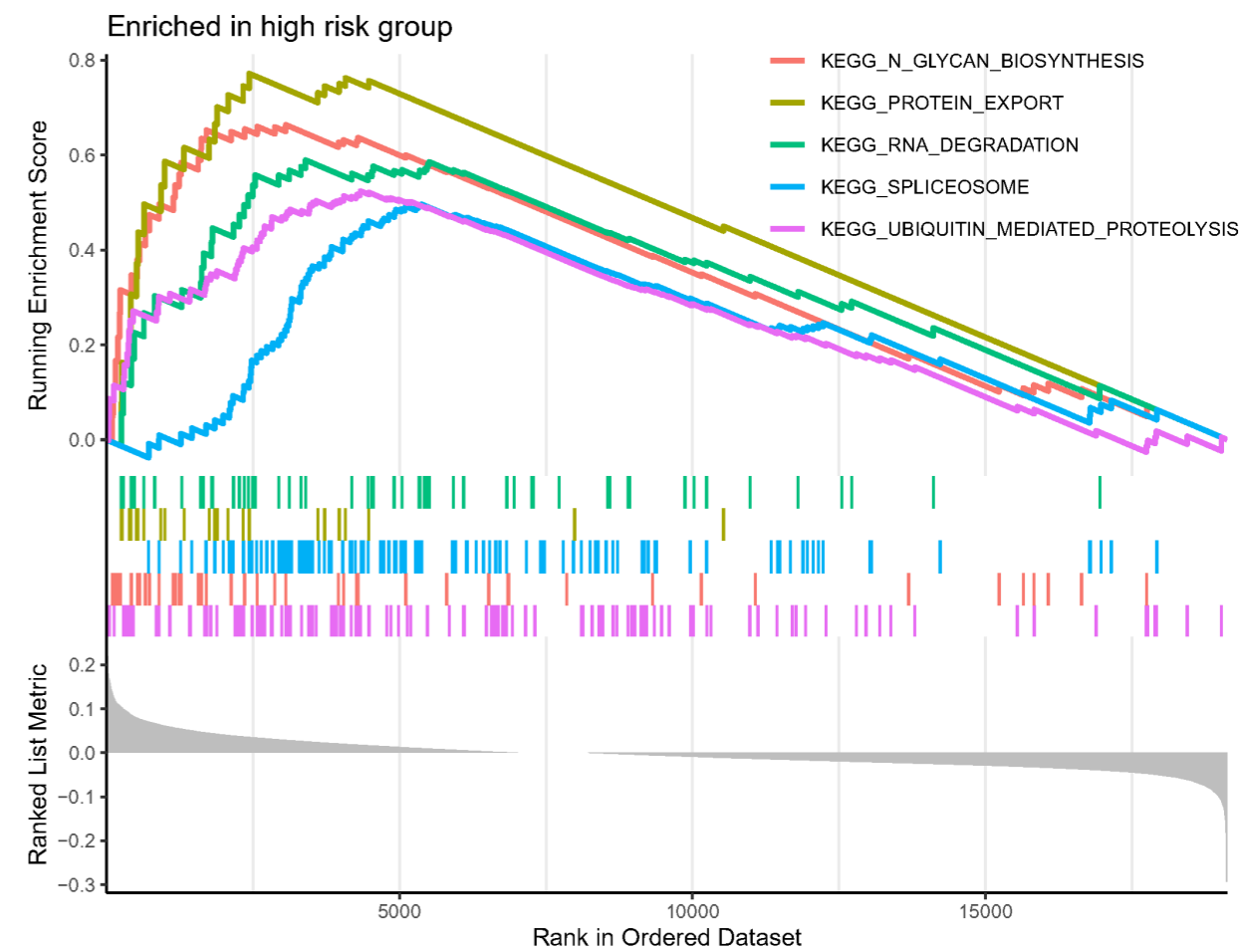

C

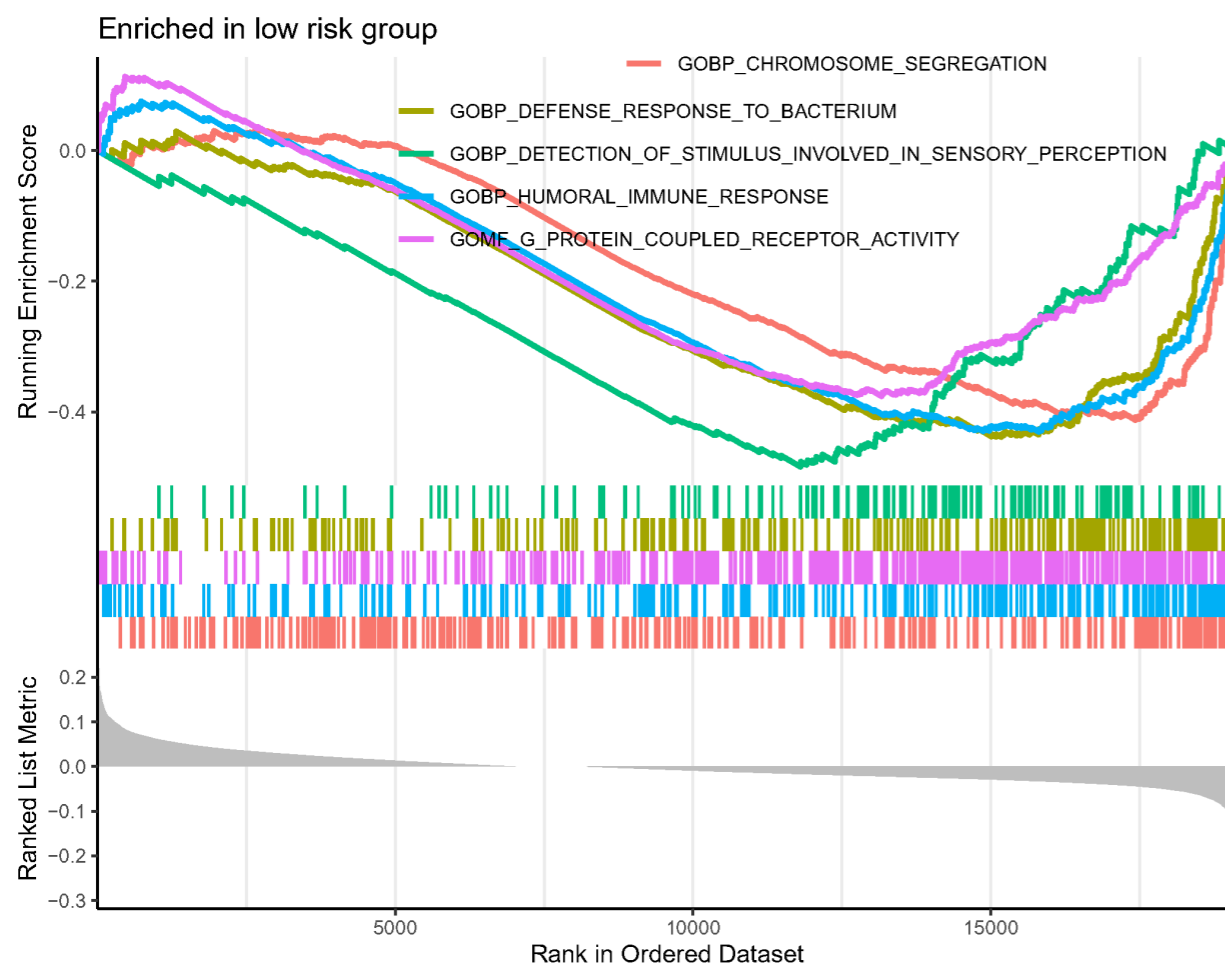

D

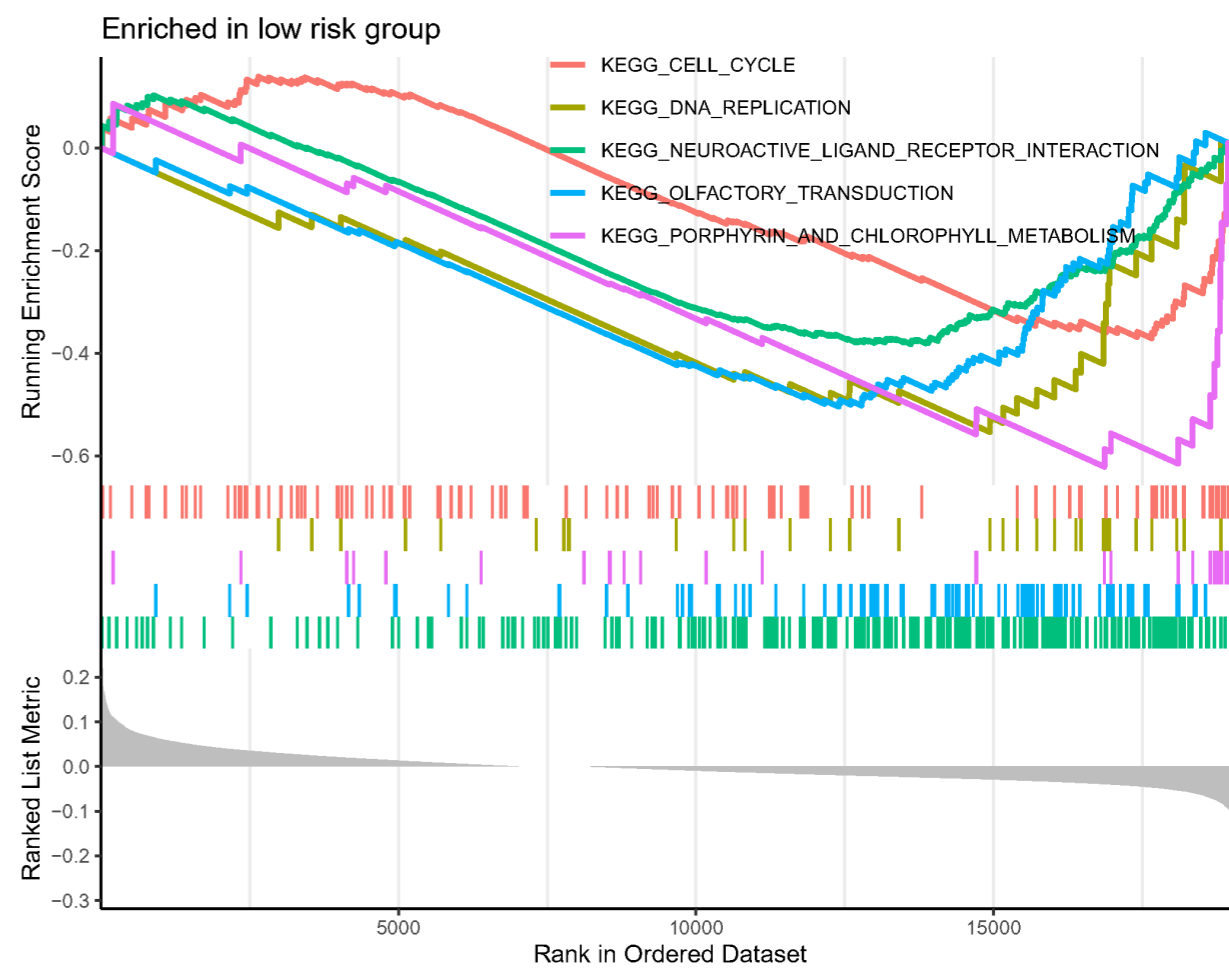

Supplement: Supplementary 3 — Supplementary Figure 3: analysis of the gene set enrichment regarding the autophagy-related signature in low- and high-risk cohorts. (a, b) Genes enriched in the high-risk cohort. (c, d) Genes enriched in the low-risk cohort. [file 3922739.f3.pdf]

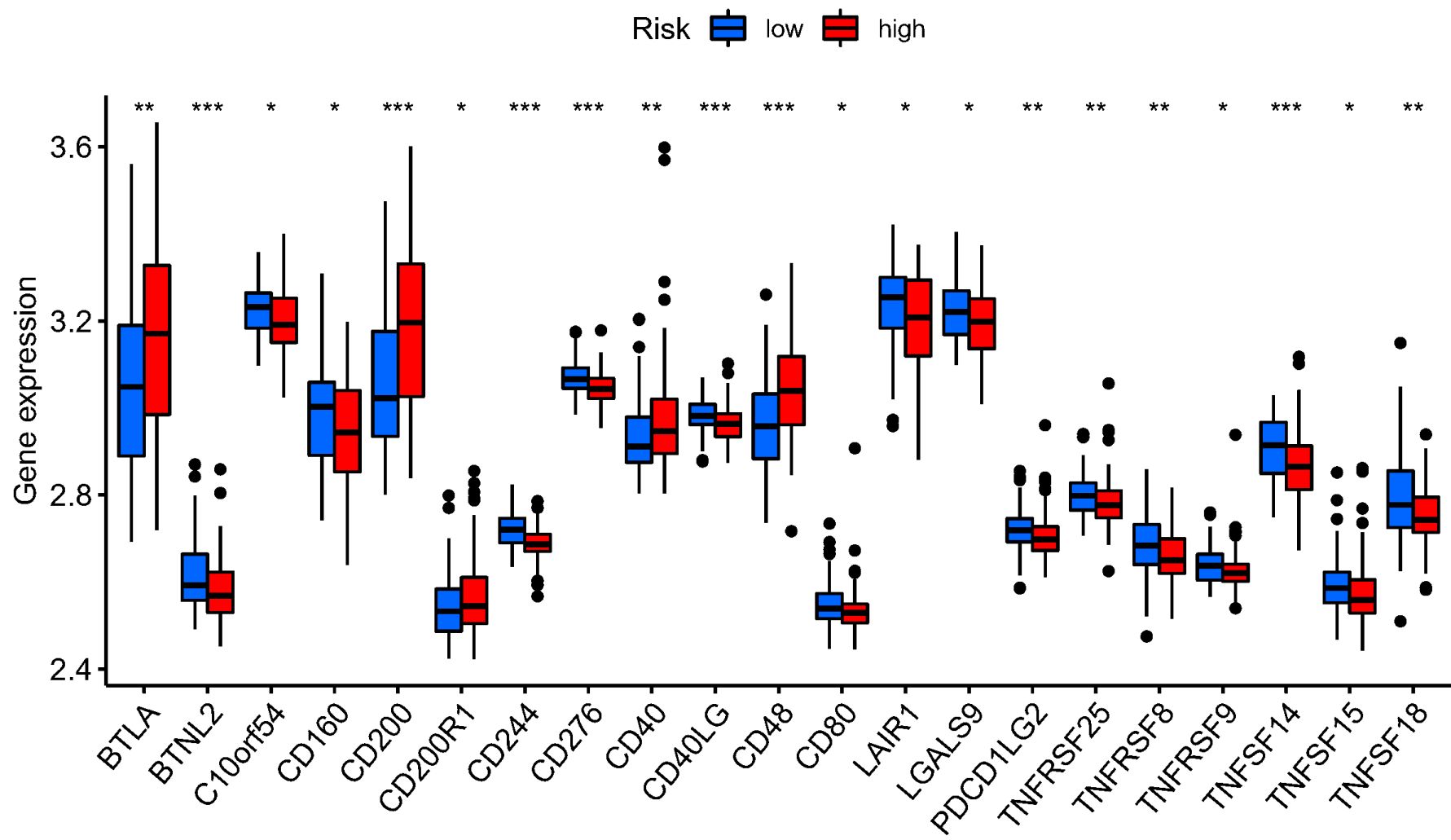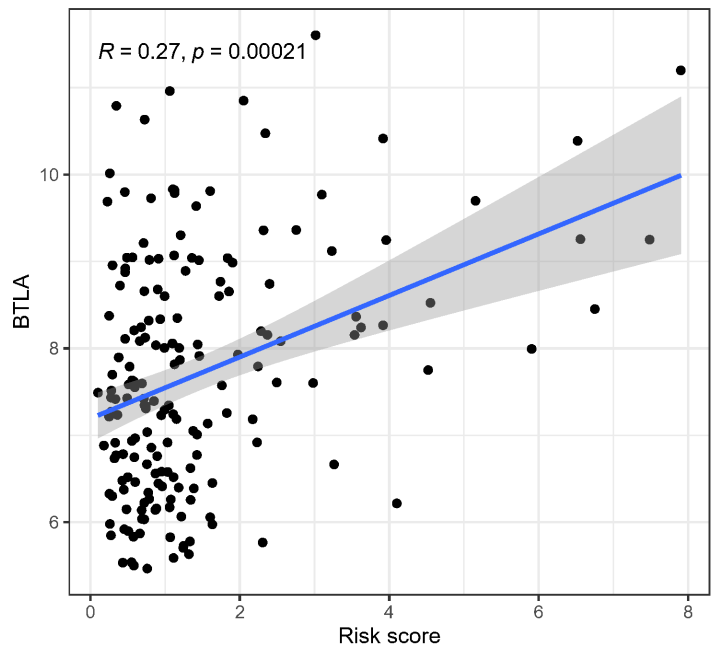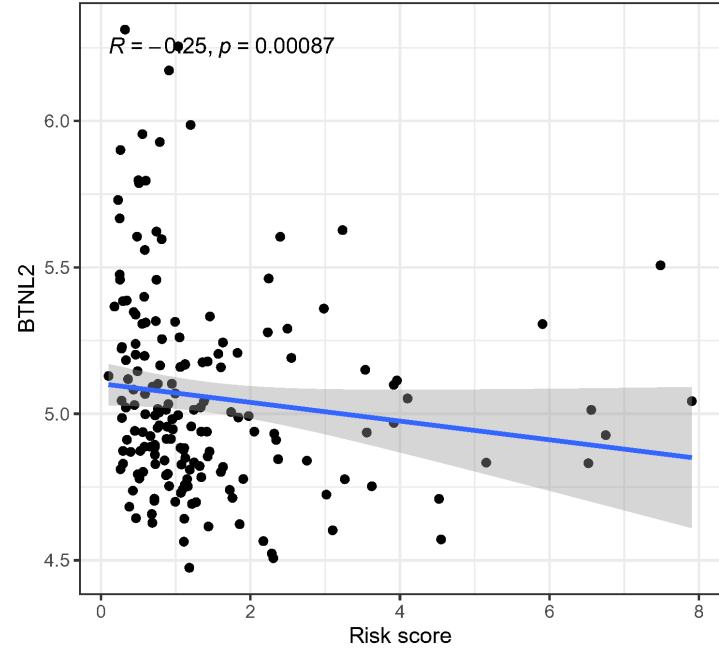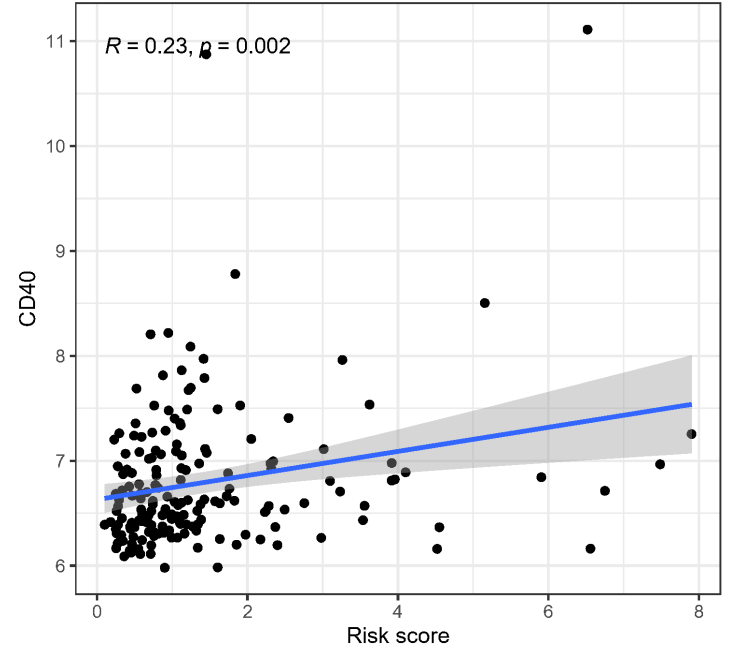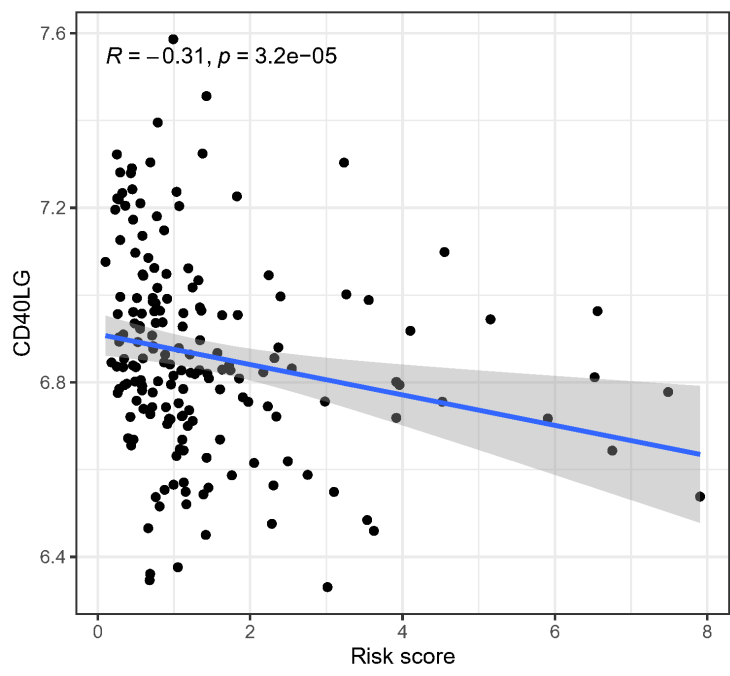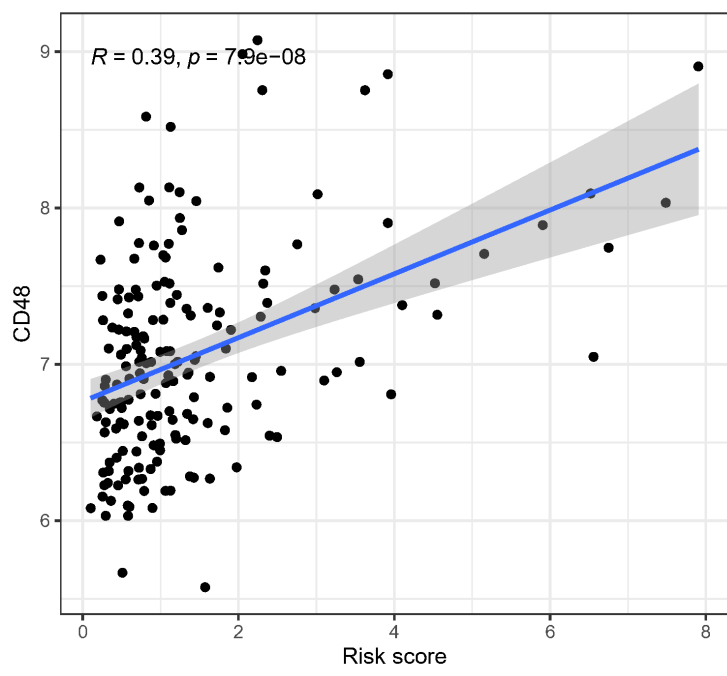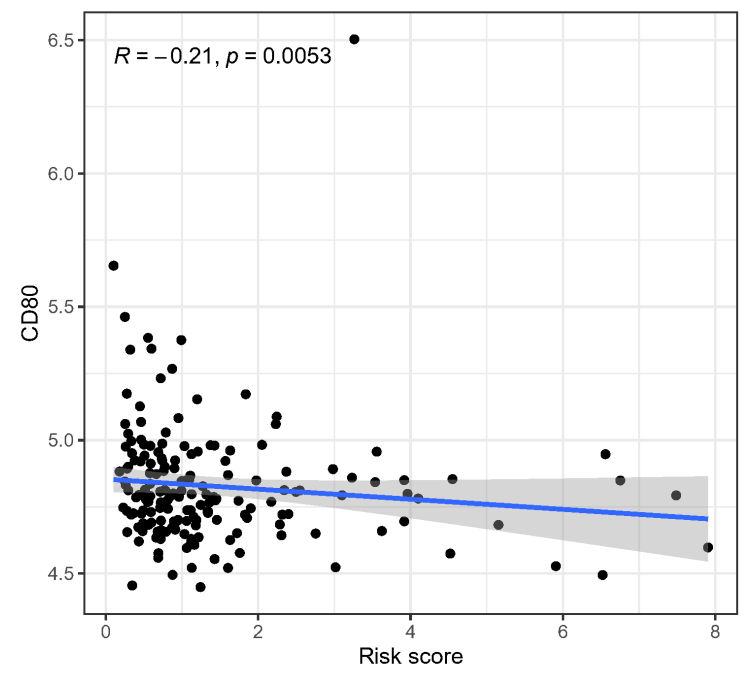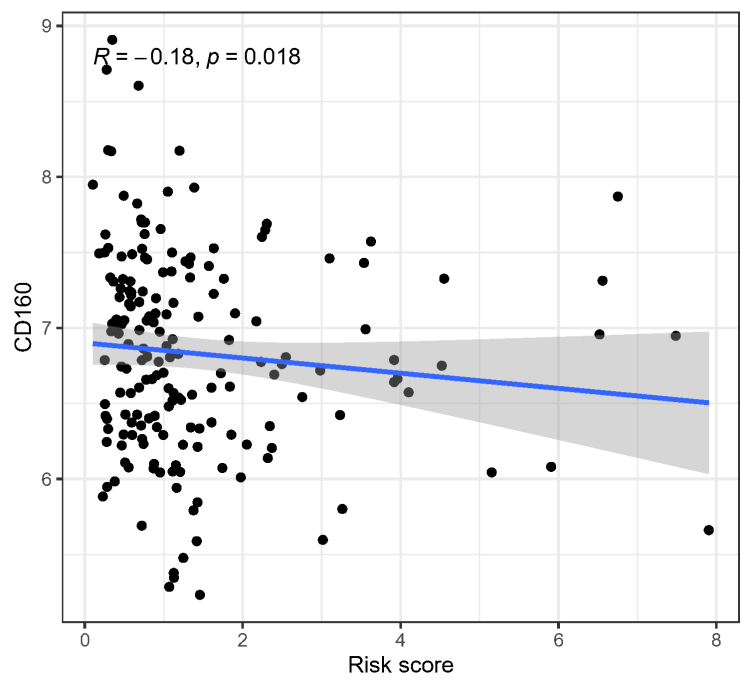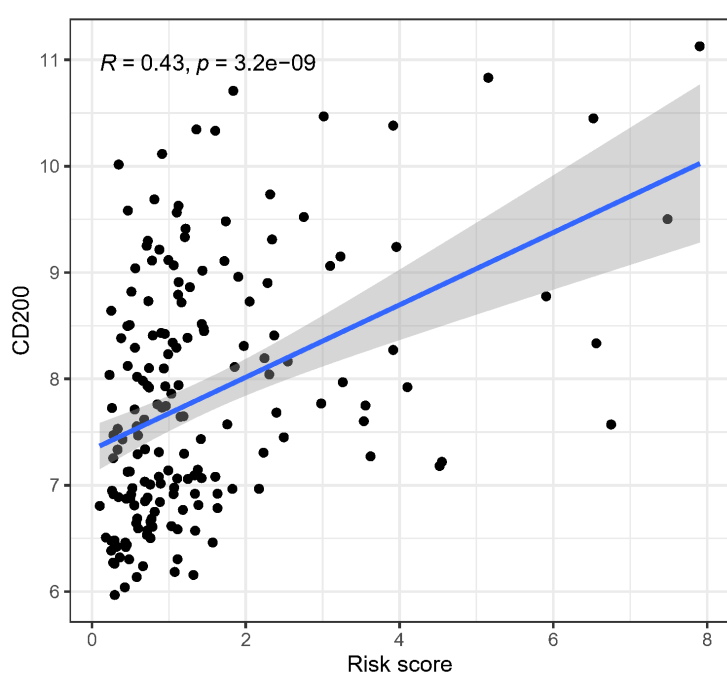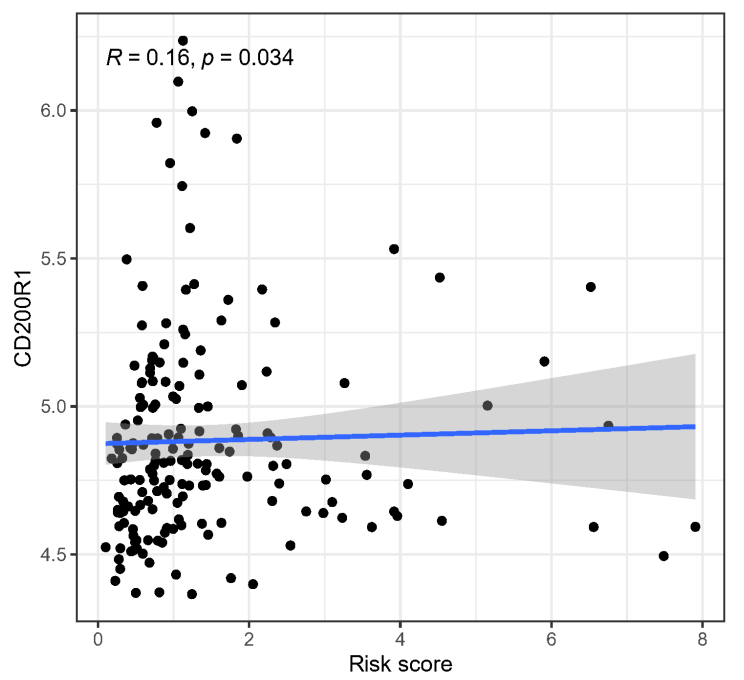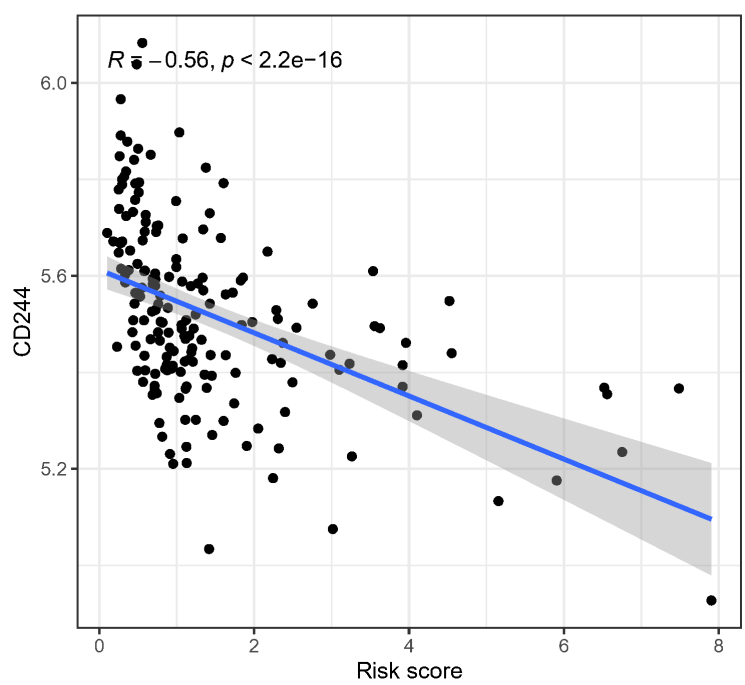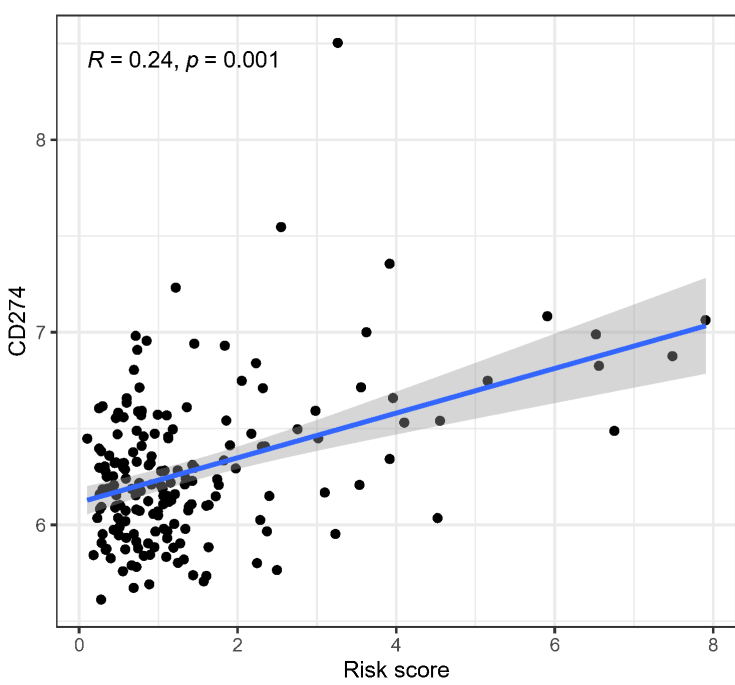

Supplement: Supplementary 4 — Supplementary Figure 4: the tumor immune microenvironment analysis in the low- and high-risk cohorts. (a) Differential expression of 21 types of immune checkpoints in the low- and high-risk cohorts. (b) Analysis of the correlation between the risk score and multiple immune checkpoints. [file 3922739.f4.pdf]
