# Supplementary material for: Identification of an Autophagy-Related Signature Based on Whole Bone Marrow Sequencing for the Prognosis and Immune Microenvironment Characterization of Multiple Myeloma
Source: J Immunol Res. 2022 May 29;2022:3922739. doi: 10.1155/2022/3922739 (PMC9169202; doi:10.1155/2022/3922739)
Supplement: Supplementary 5 — Supplementary Table 1: identification of the transcriptional regulators of OS-associated ARGs. [file 3922739.f5.docx]

**TABLE S1** | Identification of the transcriptional regulators of OS-associated ARGs.

| Key TF | P value | Q value | List of overlapped genes |
| --- | --- | --- | --- |
| PML | 0.000002 | 0.000030 | CASP3,TNFSF10,TP53 |
| TP53 | 0.000003 | 0.000030 | HSP90AB1,TP53,MYC,VEGFA,CASP3 |
| RELA | 0.000003 | 0.000030 | CASP3,VEGFA,NAMPT,MYC,TNFSF10,TP53 |
| NFKB1 | 0.000003 | 0.000030 | CASP3,NAMPT,VEGFA,TP53,TNFSF10,MYC |
| HDAC2 | 0.000007 | 0.000056 | TNFSF10,MYC,VEGFA |
| AATF | 0.000018 | 0.000109 | MYC,TP53 |
| IFI16 | 0.000018 | 0.000109 | MYC,TP53 |
| E2F1 | 0.000032 | 0.000169 | TP53,VEGFA,DAPK2,MYC |
| ING1 | 0.000038 | 0.000170 | CASP3,TP53 |
| STAT3 | 0.000040 | 0.000170 | VEGFA,CD46,MYC,TP53 |
| IRF1 | 0.000046 | 0.000171 | EIF2AK2,TNFSF10,TP53 |
| JUN | 0.000049 | 0.000171 | NAMPT,VEGFA,TP53,MYC |
| HIC1 | 0.000065 | 0.000196 | VEGFA,MYC |
| HIPK2 | 0.000065 | 0.000196 | TP53,VEGFA |
| ESR1 | 0.000153 | 0.000428 | MYC,TP53,VEGFA |
| NF1 | 0.000164 | 0.000432 | TP53,MYC |
| ING4 | 0.000190 | 0.000442 | CASP3,TP53 |
| PTTG1 | 0.000190 | 0.000442 | MYC,VEGFA |
| ErGR1 | 0.000236 | 0.000515 | TNFSF10,VEGFA,TP53 |
| FOXM1 | 0.000245 | 0.000515 | VEGFA,MYC |
| FOXO3 | 0.000276 | 0.000551 | VEGFA,TNFSF10 |
| VHL | 0.000342 | 0.000652 | TP53,VEGFA |
| HDAC3 | 0.000495 | 0.000902 | MYC,VEGFA |
| PGR | 0.000537 | 0.000902 | VEGFA,MYC |
| TCF4 | 0.000537 | 0.000902 | MYC,VEGFA |
| RUNX3 | 0.000776 | 0.001200 | CASP3,MYC |
| DNMT1 | 0.000828 | 0.001200 | VEGFA,TP53 |
| RB1 | 0.000828 | 0.001200 | MYC,VEGFA |
| SMAD3 | 0.000828 | 0.001200 | VEGFA,MYC |
| ETS2 | 0.000883 | 0.001240 | TP53,MYC |
| EZH2 | 0.001380 | 0.001810 | MYC,TP53 |
| KLF4 | 0.001380 | 0.001810 | VEGFA,TP53 |
| SIRT1 | 0.001980 | 0.002520 | MYC,TP53 |
| EP300 | 0.002690 | 0.003160 | VEGFA,MYC |
| BRCA1 | 0.002780 | 0.003160 | MYC,VEGFA |
| FOS | 0.002780 | 0.003160 | TP53,MYC |
| WT1 | 0.002780 | 0.003160 | VEGFA,MYC |
| PPARG | 0.003710 | 0.004020 | MYC,TP53 |
| SP1 | 0.003730 | 0.004020 | TNFSF10,VEGFA,MYC,CASP3 |
| YY1 | 0.006930 | 0.007280 | TP53,MYC |
| AR | 0.007230 | 0.007410 | MYC,VEGFA |
| MYC | 0.008320 | 0.008320 | VEGFA,TP53 |
