# Supplementary material for: Identification of an Autophagy-Related Signature Based on Whole Bone Marrow Sequencing for the Prognosis and Immune Microenvironment Characterization of Multiple Myeloma
Source: J Immunol Res. 2022 May 29;2022:3922739. doi: 10.1155/2022/3922739 (PMC9169202; doi:10.1155/2022/3922739)
Supplement: Supplementary 6 — Supplementary Table 2: characteristics of all patients. [file 3922739.f6.docx]

**TABLE S2** | Characteristics of all patients

| Characteristics | Values |
| --- | --- |
| Age, mean ± SD | 58.4 ± 9.0 |
| ≥ 60 | 165 (48.7) |
| < 60 | 174 (51.3) |
| Gender, n (%) |  |
| Male | 212 (62.5) |
| Female | 127 (37.5) |
| p53 | 886.2 ± 98.8 |
| > 924 | 169 (49.9) |
| ≤ 924 | 170 (50.1) |
| Mut-p53 |  |
| Yes | 127 (37.5) |
| No | 212 (62.5) |
| Reponse |  |
| SD | 25 (7.4) |
| R | 31 (9.1) |
| CR | 137 (40.4) |
| PD | 60 (17.7) |
| PR | 39 (11.5) |
| sCR | 47 (13.9) |
| Albumin, mean ± SD | 38.4 ± 6.2 |
| > 35g/L | 245 (72.3) |
| ≤ 35g/L | 94 (27.7) |
| B2m, mean ± SD | 4.8 ± 1.3 |
| > 5.5mg/L | 88 (26.0) |
| ≤ 5.5mg/L | 251 (74.0) |
| LDH, mean ± SD | 160.0 ± 76.1 |
| > 215U/L | 34 (10.0) |
| ≤ 215U/L | 305 (90.0) |
| ISS |  |
| I | 141 (41.6) |
| II | 107 (31.6) |
| III | 91 (26.8) |
| Hyper |  |
| Yes | 65 (19.2) |
| No | 274 (80.8) |
